# The challenges and strategies for navigating *in vivo* barriers to therapeutic bacterial delivery

**DOI:** 10.7150/thno.137940

**Published:** 2026-07-13

**Authors:** Haoda Yu, Jieyu Liu, Shuxin Zhang, Bing Wang, Kui Luo, Xian Jiang

**Affiliations:** 1 Department of Dermatology, Department of Radiology, Institution of Radiology and Medical Imaging, Huaxi MR Research Center (HMRRC), Department of Critical Care Medicine, West China Hospital, Sichuan University, Chengdu, Sichuan 610041, China.; 2 Functional and Molecular Imaging Key Laboratory of Sichuan Province, NHC Key Laboratory of Transplant Engineering and Immunology, Research Unit of Psychoradiology, Chinese Academy of Medical Sciences, Chengdu 610041, China.

**Keywords:** bacterial therapy, drug delivery, therapeutics, biological barriers, probiotics

## Abstract

Bacteria have emerged as promising living therapeutics for the treatment of infections, inflammatory disorders, metabolic diseases, and neoplastic diseases. However, their clinical efficacy is often compromised by *in vivo* barriers that reduce their viability and impair their function after administration. Rational engineering can help them sustain their viability under a harsh microenvironment and enhance their targetability as well as retention, ultimately improving their functional performance *in vivo*. This review starts with a concise synopsis of current status in live bacterial therapy, with particular emphasis on the major biological barriers to their *in vivo* application. Engineering strategies for harnessing bacterial intrinsic characteristics and overcoming their specific barriers are discussed. Representative examples are then presented to illustrate how engineered live bacteria overcome these biological barriers and achieve therapeutic applications via oral, topical, intravenous, and inhalational administration. Finally, we identify the key challenges associated with translating laboratory advances into reliable and safe clinical therapeutics.

## 1. Introduction

**Figure 1:**
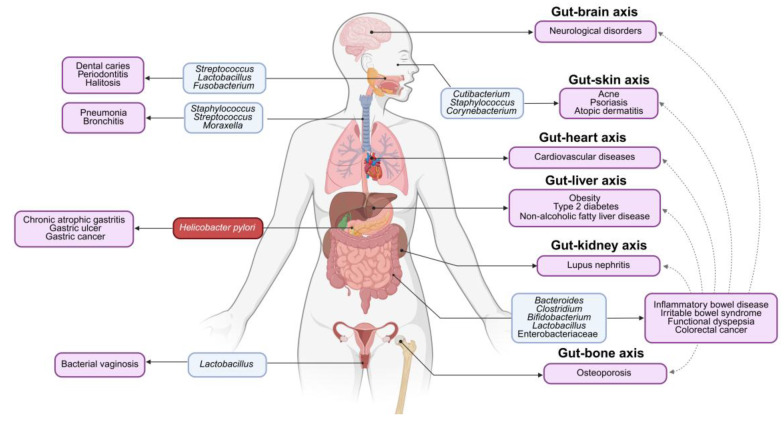
Schematic diagram for representative examples of bacteria and their associated diseases. Notably, the gut microbiota can induce various syndromes in distant organs via the gut-organ axes, such as the gut-brain, gut-skin, gut-heart, gut-liver, gut-kidney or gut-bone axis. Created in https://BioRender.com.

An adult human body hosts estimated 38 trillion bacteria, exceeding 30 trillion human cells within the body. Increasing evidence has seen microbial dysbiosis as a driving force to pathogenesis of metabolic disorders, inflammation, cancer, and infections 1 (**Figure 1**). The therapeutic use of bacteria can be traced back to over a century ago, when Tissier isolated *Bifidobacterium* from the feces of infants and observed its association with reduced incidences of diarrhea in 1889. In this context, bacteria have been widely investigated as promising living therapeutics for disease treatment because they can produce bioactive metabolites and modulate immunity 2. Moreover, bacteria can be used as drug delivery systems to hypoxic lesions, such as tumor tissues 3, 4. To note, bacterial therapeutics are used as a broad term in this review, including probiotic bacteria, commensal bacteria, genetically engineered bacteria and attenuated bacteria.

Despite their encouraging therapeutic promise, the efficacy of exogenously administered bacteria is significantly impacted by multiple biological barriers encountered during delivery and after administration. Oral bacteria must survive gastric acidity, oxidative stress, intestinal peristalsis, competition from resident microbiota, and, in some cases, concurrent antibiotic exposure 5. Topically delivered bacteria are exposed to local hostile physicochemical conditions 6. Intravenously administered bacteria are subjected to complement activation, phagocytic clearance, and insufficient accumulation at target lesions 7, and inhaled bacteria must withstand formulation-associated stress and evade mucociliary clearance. Collectively, these route-dependent biological barriers impose major constraints on the *in vivo* performance of therapeutic bacteria, thereby motivating the development of engineering strategies to improve their viability, persistence, targeting, and therapeutic efficacy 8.

Engineering bacteria provides a potent avenue for enhancing the therapeutic efficacy by improving bacterial tolerance to hostile *in vivo* environments and endowing them with protective, targeting, and therapeutic functions beyond their native capacity. The integrated engineering approaches hold promises for addressing multiple barriers encountered by bacteria during the courses of their delivery and therapeutic action. However, effective bacterial engineering relies on the successful incorporation of functional moieties onto bacterial cells, as well as the mechanistic action of the bacterial chassis. The structural, physicochemical, and biosynthetic attributes of bacteria offer a set of engineerable interfaces, and these interfaces can be selectively exploited for precise moieties coupling. More importantly, these engineerable interfaces allow rational alignment of exogenous functions with the native biological activities of bacterial cells, achieving context-specific therapeutic synergy and enhancing therapeutic efficacy.

In this review, we provide an overview of engineering strategies to improve bacterial delivery efficiency and enhance therapeutic efficacy. Importantly, we elaborate on the rationale behind employing these strategies to overcome the *in vivo* biological barriers that impact bacterial performance. This review initially introduces bacterial structural and biological features. By harnessing specific structural, physicochemical, or biological characteristics of bacteria, engineering strategies can be systematically classified according to the specific bacterial characteristics they exploit. Major categories include surface engineering based on bacterial physicochemical properties, covalent or dynamic surface functionalization enabled by exposed reactive groups, biosynthesis-expanded surface engineering that introduces non-native modification handles through endogenous bacterial processes, and strategies involving intracellular cargo loading or bulk encapsulation. We delve into functional tailoring of these strategies to address the significant biological barriers that bacteria encounter during oral, topical, intravenous, and inhalational delivery, such as physicochemical stress, inadequate adhesion, nutritional constraints, microbial competition, immune and antibiotic clearance, and insufficient site-specific targeting. We then survey therapeutic application examples of engineered bacteria via different administration routes. Finally, we discuss principal translational challenges and propose future directions for the development of next-generation bacterial therapeutics.

## 2. Foundations for bacterial therapeutic engineering

### 2.1 Biological foundations of bacterial therapeutics

Resident bacteria in the human body confer health benefits through multiple mechanisms. The development of bacterial therapeutics starts with the selection of appropriate bacterial chassis from the resident bacterial community to confer therapeutic benefits. Because different bacterial species vary in their colonization niches, oxygen preferences, and tissue-tropic behaviors, the selection of bacterial species should be aligned with the target organ or the disease microenvironment to achieve effective and feasible bacterial therapy. For example, in the context of inflammatory bowel disease (IBD) characterized with dysbiotic gut microbiota, probiotic and commensal bacteria, such as *Lactobacillus*, *Bifidobacterium*, *Escherichia coli Nissle 1917* (EcN), and *Clostridium*, contribute to disease resolution through four distinct mechanisms 9. First, they engage in direct competition with pathogens for the nutrients available and adhesion sites on the intestinal mucosa 10. Second, bacteria suppress pathogen proliferation via producing a range of biologically active metabolites including bacteriocins, antimicrobial peptides (AMPs), and lactic acid. Third, they maintain the integrity of the intestinal epithelium by upregulating the expression of tight junction proteins, stimulating mucus secretion by intestinal epithelial cells, and promoting the secretion of short-chain fatty acids (SCFAs). Finally, bacteria can help resolve inflammation and neutralize reactive oxygen species (ROS) through inducing the differentiation of regulatory T cells.

The skin is colonized by diverse microbial communities. Strikingly, mutually beneficial bacteria play a critical role in training the skin immune system to resist the invasion of pathogenic substances. For instance, *Staphylococcus epidermidis* offers protection to the host against skin infections through activating CD8^+^ T cells and exciting keratinocytes to discharge AMPs 11. In addition, Staphylococcal lipoteichoic acid can maintain the integrity of epithelium, and facilitate tissue repair through alleviating inflammation by blocking the Toll-like receptor 2 (TLR2)-associated pathway 12. In conclusion, skin microbiota plays an indispensable role in maintaining epithelial homeostasis and overall skin health.

The tumor microenvironment (TME) is an immunosuppressive milieu characterized by hypoxia, abundant immunosuppressive metabolites, and deprived nutrients, which collectively diminish the efficacy of immunotherapy 13-17. Recent studies have revealed that gut microbiota can stimulate host immunity and act in synergy with cancer immunotherapy for anticancer treatment. For instance, *Lactobacillus reuteri* and *Lactobacillus plantarum* have been shown to activate neutrophils and natural killer (NK) cells, thereby promoting pro-inflammatory cytokine production within the TME and inhibiting tumor progression 18. Furthermore, dendritic cells (DCs) can be activated upon recognizing bacterial components, such as lipopolysaccharide and peptidoglycan, subsequently recruiting effector T cells to eliminate tumors 19. Notably, the abundance of *Bifidobacterium* and *Enterococcus* in the feces of melanoma patients is positively correlated with the therapeutic response to anti-PD-L1 antibodies 20. However, intratumoral bacteria are found to interact with immune cells and promote tumor progression 21. For example, *Fusobacterium nucleatum* residing in colorectal cancer can protect tumor cells from the elimination by cytotoxic NK cells via various means 22. Therefore, bacteria can be administered to regulate the population of intratumoral immune cells and activate their anti-tumor immune responses, offering new insights into cancer immunotherapy.

Although bacteria exhibit multiple therapeutic mechanisms, their effective delivery to target sites is often hindered by a myriad of biological barriers. In this section, we summarize the application of bacteria for a variety of indications, with a particular focus on those native or genetically engineered bacteria that have entered clinical trial phases (**Table 1**). We then introduce bacterial delivery challenges including oral, topical, intravenous and inhalational administration.

### 2.2 Administration route-specific barriers

Oral administration is the most conventional and widely accepted route for bacterial therapy. When bacteria enter the body, they are exposed to a series of harsh gastrointestinal microenvironments. Gastric fluid is the first crucial biological barrier. Gastric acid can significantly reduce the survival rate of bacteria through breaking the integrity of microbial cell membranes and inducing protein denaturation 23. After the bacteria survive through the stomach, they enter an alkaline environment of the small intestine. Bile salts and digestive enzymes serve as the second barrier. Bile salts can penetrate the phospholipid double layer of the bacterial cell membrane, leading to the damage of cell membranes and the disturbance of the mechanisms of DNA repair, ultimately reducing bacterial survival and preventing bacterial growth 24. It is noted that bacteria must achieve context-dependent optimal colonization within the gut to bring sustained therapeutic benefits. Unfortunately, the administered bacteria may be expelled by the originally resident microorganism group, because these bacteria compete for nutritional metabolites as well as attachment sites at the intestine site with resident bacteria. In addition, they may not colonize successfully at the intestine site due to rapid intestine movements 25. It is worth noting that the intestinal micro-environment of colitis displays the characteristics of excessive oxidative stress and inflammatory reaction, which can inhibit the colonization, survival, and growth of bacteria 26. Once bacteria successfully establish themselves in the intestine tract and proliferate to an adequate quantity, they can commence their therapeutic function.

Bacterial therapy can be topically applied to treat skin and mucous membrane diseases by the restoration of the microbial population balance at these sites. Nevertheless, physical and biological barriers may directly impact the restoration process, thus diminishing their therapeutic efficacy 27. Physical and chemical properties at these sites, including local pH, water moisture, and surface chemistry, may reduce bacterial viability and impair their metabolic activity 6. In addition, the resident microbial groups in the host prevent the applied bacteria from occupying ecological niches by competing for nutrients and attachment positions 28. For example, these resident bacteria may form thick harmful biofilms, and these biofilms act as an extra physical obstacle to block the approach of bacteria to the surface of the skin tissue, obstructing adherence and inhabitation of topically applied bacteria 1. Furthermore, the host inflammation reaction adds one more layer of complexity. The reaction may lead to the formation of a micro-environment rich in cytokines and ROS, which can damage bacterial membranes and weaken the survival of bacteria 29. The restoration process by topical application of bacteria may be significantly impeded when antibiotics are used for infection control. The exposure to antimicrobial drugs can kill pathogenic bacteria at these sites; however, this may eliminate beneficial resident microbial groups as well as bacteria, thus reducing the likelihood of prolonged and steady settling of bacteria after the treatment 30.

Intravenous route of bacterial therapy presents an advantage of bypassing gastrointestinal barriers. After administration, they directly enter the circulatory system, thus enhancing their delivery efficiency. However, this route is impeded by host immune clearance. The mononuclear phagocyte system serves as the primary mechanism to eliminate circulating foreign bacteria 31. Besides, the activated complement system can directly lyse bacteria through the formation of the membrane attack complexes 32. Since systemic administration of billions of bacterial spores inevitably leads to septicaemia 7, it is crucial to ensure the intravenously administered bacteria can target lesion sites and maintain their biological function on these sites.

The respiratory tract is directly exposed to an external environment, and it displays a branching architecture with regionally distinct deposition patterns, creating formulation and translational challenges for local delivery of living bacteria. To reach the targeted pulmonary region, inhaled bacteria must be delivered as aerosols, and the aerodynamic particle size of aerosols is critical for their efficient deposition because of variations in the airway caliber and airflow throughout the bronchial tree 33. The bacterial viability and function must be maintained during preparation and administration of aerosols. Nebulization of liquid formulations may alter the bacterial population 34, and the preparation of dry powders introduces dehydration and processing stresses that may lead to a reduction in bacterial viability 35. After delivery of aerosols, clearance through mucus or mucociliary structures and alveolar phagocytes may diminish bacterial persistence in the targeted region and compromise their therapeutic activity 36. Moreover, chronic lung diseases are often accompanied with tissue injury, sustained inflammation and local microbial dysbiosis 37. Therefore, interactions between administered bacteria and this altered pulmonary microenvironment may provoke unintended inflammatory responses, and extensive safety evaluation should be performed during clinical translation. Furthermore, excessive ROS in the diseased pulmonary microenvironment may impair the bioactivity of administered probiotic bacteria, thereby reducing their therapeutic efficacy 38.

Collectively, unsatisfied therapeutic efficacy of exogenous bacteria may be attributed to their low viability as well as their susceptibility to ecological stress due to physicochemical/biological barriers at the target site. Therefore, these microorganisms before administration can be engineered to improve overall fitness and stress tolerance, ultimately enhancing their therapeutic performance 39.

## 3. Engineering bacteria to overcome *in vivo* barriers

**Figure 2:**
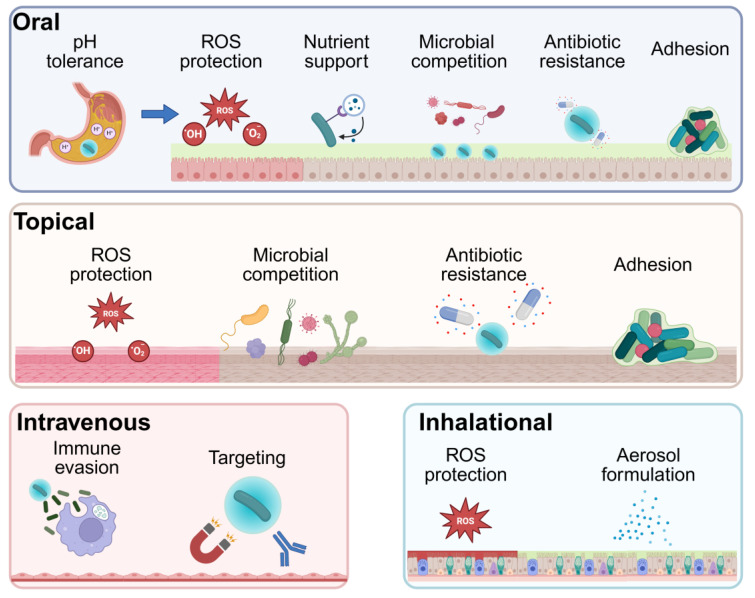
Representative route-specific engineering strategies of therapeutic bacteria. Therapeutic bacteria encounter distinct biological barriers depending on the route of administration. During oral delivery, engineered coatings or encapsulation systems can protect bacteria from gastric acidity, reactive oxygen species (ROS), nutrient limitation, microbial competition, antibiotics, and weak mucosal adhesion, thereby improving survival and intestinal retention. For topical delivery, bacteria must tolerate oxidative stress, compete with resident or pathogenic microorganisms, survive after local antibiotic exposure, and adhere to skin or mucosal surfaces. Intravenously administered bacteria face rapid immune clearance and immune-evasive modification, or active targeting strategies can be used to improve circulation persistence and lesion accumulation. For inhalational delivery, bacteria should be formulated into aerosols, in the format of respirable droplets or dry powder particles, for effective deposition in the bronchial and small airway regions. In addition, ROS-scavenging biomaterials can protect bacteria from oxidative stress in inflamed lung tissues and help maintain their therapeutic activity. Created in https://BioRender.com.

The curative effect of administered bacteria on reshaping the microbe community and treating a broad spectrum of diseases has been extensively documented. However, bacteria which are administered without engineering manipulation often have poor survival *in vivo*, because they face harsh microenvironmental conditions, including acidic pH, excess ROS, poor adhesion, and weak niche competition with existing microbial groups on the disease site. For solving these shortcomings, many kinds of surface engineering methods have been studied to raise the survival ability and biological usability of treatment bacteria when compared with their not modified original types (**Figure 2**). Accordingly, this section highlights the major engineering approaches designed to overcome the challenges encountered by administered bacteria. The ability of engineered bacterial therapeutics to overcome *in vivo* biological barriers and achieve enhanced therapeutic efficacy predominantly depends on the materials integrated with bacterial cells. These materials can protect bacterial viability under harsh physiological conditions, improve resistance to immune clearance and external stresses, and enhance bacterial adhesion and targeting capabilities. As a result, material engineering plays a critical role in improving the *in vivo* performance of bacterial therapeutics.

### 3.1 pH shielding

The pH value in an environment plays a crucial role in the survival and function of live bacterial therapeutics. Exposure of them to unfavorable pH can damage their membranes, disrupt their metabolic activity, and reduce their viability, thereby substantially diminishing the proportion of administered bacteria that retain their functional efficacy at the target site. This challenge is especially associated with the oral delivery route. In this route, a strongly acidic gastric environment represents a major barrier. A crucial aspect in bacterial engineering is safeguarding them against acid-induced damage.

**Figure 3:**
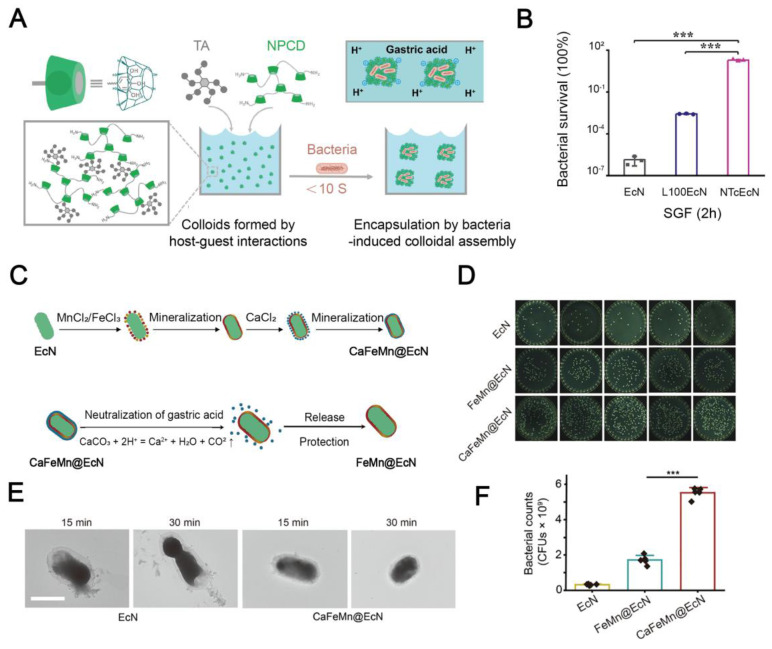
Representative examples of pH-protective engineering strategies. (A) Schematic of colloidal encapsulation of bacteria to enhance their gastric acid resistance during oral delivery. (B) Survival of NTcEcN, naked EcN, and L100EcN after exposure to a simulated gastric fluid for 2 h. Adapted with permission from 46, copyright 2023, American Chemical Society. (C) Diagram for preparing theranostic CaFeMn@EcN via *in situ* sequential mineralization. The outer CaCO_3_ layer neutralizes gastric acids to protect the activities of the encapsulated bacteria. (D) Photographs of culture plates, (E-F) transmission electron microscopy (TEM) images and the survival rate of EcN, FeMn@EcN, and CaFeMn@EcN after 30-min exposure to a simulated gastric fluid. Adapted with permission from 48, copyright 2025, The American Association for the Advacement of Science.

An array of coatings have been designed to shield bacteria from the gastric environment and dissolve upon entry into the intestine, including Eudragit L100-55, a clinically used polymer coating 40, 41, chitosan/sodium alginate coatings 42, two-dimensional nanosheets 43, and polyelectrolyte composite coatings 44. Notably, a polyelectrolyte complex formed via electrostatic and hydrogen-bonding interactions between oxidized starch and polyethylenimine enhanced the survival of EcN by approximately 40-fold in a simulated gastric fluid and 74-fold in an intestinal fluid 25. Encapsulation of bacteria within biomaterial matrices, such as colloids and hydrogels, is another effective strategy to shield them from the gastric fluid 45, 46. Of particular interest are positively charged colloids composed of tannic acid and poly-β-cyclodextrin, which electrostatically encapsulated bacteria and formed a thick protective shell. Up to 19% of the bacteria inside the shell remained viable in a simulated gastric fluid, and the value is 7,500 times higher than that of Eudragit L100-55 46 (**Figure 3A-B**). Apart from physically shielding bacteria from gastric acid, protons can be obtained during engineering bacteria shielding can also seize protons to provide acidic protection. For example, EcN was coated with carboxymethylated β-glucan (mGN), conferring a 1720-fold survival rate in a simulated gastric fluid compared to the bare counterpartner, which may be attributed to the capture of H⁺ by -COO^-^ of gastric acids 47. However, simulated gastric and intestinal fluids cannot recapitulate the complexity in the gastrointestinal environment, including mucus diffusion, peristalsis, bile and enzyme dynamics, microbiota competition, dietary effects, or disease-related changes in the gut. Therefore, improved survival rates in the simulated fluids should be viewed as the evidence to support stress tolerance, rather than *in vivo* colonization or therapeutic efficacy.

Mineralization offers an alternative acid-protective approach by neutralizing excess acids in a gastric environment. A mineral layer composed of MnO_2_ and Fe_2_O_3_ nanoparticles was first deposited on the bacterial surface, and the inner layer served as a substrate for the sequential growth of an outer CaCO_3_ shell. The CaCO_3_ shell neutralized gastric acids and preserved the bacterial viability 48 (**Figure 3C-F**). Collaboratively, engineered bacteria with acid resistance may survive in a harsh stomach environment and colonize the intestinal tract.

### 3.2 ROS protection

Oxidative stress is a well-established feature in inflammatory diseases, such as IBD and chronic wounds. Excessive ROS contributes to epithelial injury, inflammatory amplification and impaired tissue repair. This redox-hostile milieu compromises the viability and bioactivity of bacteria at diseased sites, thereby diminishing their colonization and reducing their therapeutic persistence 49, 50. Therefore, it is a valuable therapeutic strategy to engineer bacteria with the capacity for withstanding oxidative stress. The widely used ROS-scavenging strategies can be roughly divided into direct ROS consumption and catalytic ROS scavenging 51.

**Figure 4:**
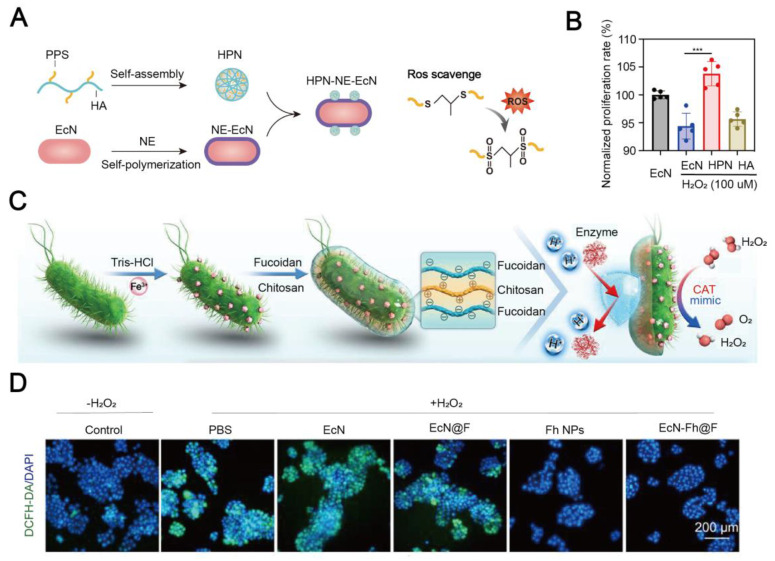
Representative examples of ROS-scavenging engineering strategies. (A) Diagram for preparing HPN-NE-EcN and its ROS-scavenging activity by oxidizing sulfur atoms to form sulfoxides and then sulfones. (B) The normalized proliferation rate of EcN after culture in the LB medium containing H_2_O_2_ with or without HPN treatment. Adapted with permission from 52, copyright 2022, The American Association for the Advancement of Science. (C) Schematic illustration of preparing antioxidative engineered EcN-Fh. (D) The ROS-scavenging activity of EcN-Fh@F *in vitro*. Adapted with permission from 57, copyright 2024, American Chemical Society.

One approach for direct ROS consumption is to coat bacteria with ROS-responsive organic materials. For example, Zhang *et al.* decorated EcN with hyaluronic acid-poly(propylene sulfide) nanoparticles. Poly(propylene sulfide) acted as a ROS-consuming component because its sulfur atoms can be readily oxidized by ROS 52 (**Figure 4A-B**). This design enabled direct ROS scavenging and protected the bacteria against oxidative damage in an inflammatory intestinal environment. Another example for direct ROS consumption is the employment of lipid coatings loaded with antioxidant compounds, such as pterostilbene or astaxanthin. These compounds react with ROS to mitigate oxidative damage and improve bacterial persistence in the presence of a high level of ROS 53, 54.

Nanozymes have attracted considerable interest in engineering bacteria because their catalytic nature endows them with sustained ROS-scavenging capability. Mechanistically, SOD-like nanozymes catalyze the dismutation of superoxide anions into H_2_O_2_ and O_2_, whereas CAT-like nanozymes decompose H_2_O_2_ into H_2_O and O_2_ 55. Diverse nanozyme systems, including metal-based nanozymes, carbon-based nanozymes, and single-atom nanozymes, have been developed for bacterial engineering to eliminate excess ROS at inflammatory sites and generate a habitable microenvironment for bacterial colonization 56-60. For example, ferrihydrite nanoparticles were anchored onto EcN to construct EcN-Fh, which exhibited CAT-like activity. The CAT-like nanozymes reduced the H_2_O_2_ content by 80% within 12 h 57 (**Figure 4C-E**). This activity was attributed to the Fe³⁺-rich oxyhydroxide interface of ferrihydrite, where surface iron-associated hydroxyl groups and adjacent Fe sites cooperatively activated H_2_O_2_ and promoted its decomposition into H_2_O and O_2_ 61. More recently, hybrid coatings have been developed by integrating direct ROS consumption with catalytic ROS elimination. Liang *et al.* reported a biocompatible polydopamine-based nanozyme shell. The catechol groups on the shell enabled direct radical scavenging and cerium oxide provided CAT-like activity. The resulting nanozyme shell for probiotics (EcN@DMCe) rapidly decomposed H_2_O_2_ into O_2_ at a H_2_O_2_ concentration of 10 mM and oxygen generation reached an equilibrium state within 10 min 62, as the defects in the crystal lattice and oxygen vacancy-coupled Ce^3+^/Ce^4+^ cycling facilitated H_2_O_2_ adsorption and decomposition into H_2_O and O_2_ 63.

### 3.3 Adhesion enhancement

**Figure 5:**
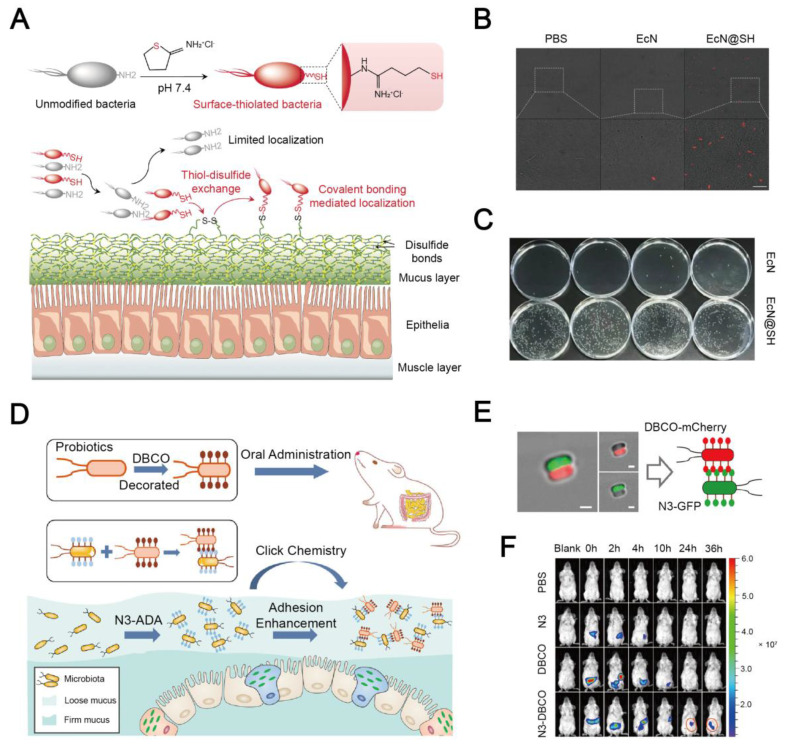
Representative examples of adhesion-enhancing engineering strategies. (A) Schematic illustration of preparing surface-thiolated bacteria that can be chemically bound to poly(disulfide)s-abundant mucin via catalyst-free dynamic thiol-disulfide exchange reaction. (B) Representative confocal laser scanning microscopy (CLSM) images of the mouse mucus layer. (Scale bar: 10 µm). (C) Typical digital images of agar plates containing EcN collected from mouse jejunal mucus. Adapted with permission from 67, copyright 2022, Springer Nature. (D) Schematic illustration of colonization enhancement for delivered bacteria through biorthogonal conjugation with gut inhabitants via click chemistry. (E) CLSM images of bacterial adhesion between N3-GFP and DBCO-mCherry. (Scale bar: 1 µm). (F) Representative *in vivo* imaging system (IVIS) images for evaluating bacterial retention in the gastrointestinal tract of mice. Adapted with permission from 68, copyright 2022, American Chemical Society.

Effective adhesion and prolonged retention at the target site are critical for bacterial efficacy. Competitively occupying the target site by administered bacteria can prevent their clearance, sustain the abundance of the local bacterial population, and promote intimate host–microbe interactions. Securing spatial niches at the target site also supports pathogen exclusion, thereby prolonging the therapeutic activity of bacteria 64, 65. However, disease-associated mucosal damage, together with persistent peristalsis and luminal washout, could markedly accelerate bacterial clearance. To address these challenging issues, multiple strategies have been developed to enhance the adhesion and colonization of engineered bacteria 65-68. For instance, conversion of primary amino groups on the bacterial surface into free thiols enables the formation of covalent bonds between bacteria and disulfide-rich mucin through catalyst-free thiol-disulfide exchange 67. The number of engineered bacteria colonizing jejunal mucus was 171.3 times higher than native bacteria (**Figure 5A-C**). Tannic acid-incorporated coatings can also improve intestinal mucosal retention by 2.5-fold through hydrogen-bond-mediated interactions and scavenge excessive ROS under pathological conditions 66. In addition to direct mucus binding, bacterial colonization can be directed toward specific local niches by exploiting interactions with the bacteria that are established at the target site. For example, incorporation of azido-modified D-alanine into peptidoglycan of gut-resident bacteria facilitates bioorthogonal anchoring of DBCO-modified *Clostridium butyricum*, thereby extending the adhesion and colonization time at the target intestinal site to 48 h 68 (**Figure 5D-F**).

### 3.4 Nutrient support

**Figure 6:**
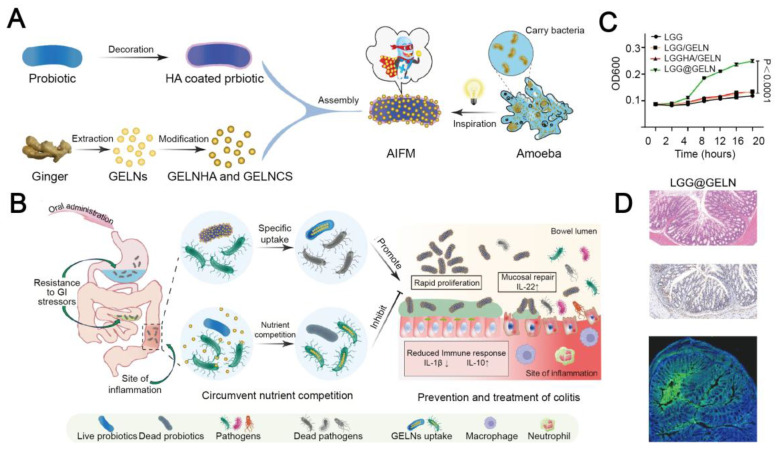
Representative examples of nutrient-supplying engineering strategies. (A) Schematic illustration of the fabrication of nutrient-carrying microecologics (AIFM). (B) Diagram for enhanced survival, proliferation, and colonization of bacteria through specific uptake of prebound nutrients from AIFM. (C) The proliferation of *L.reuteri* in a simulated nutrient-deprived intestinal fluid without the presence of pathogens. (D) The therapeutic efficacy of LGG@GELN in dextran sulfate sodium (DSS)-induced colitis. Adapted with permission from 71, copyright 2025, Springer Nature.

Bacteria, as living therapeutics, require adequate nutritional support to sustain their viability, metabolic activity, and functional persistence after administration. Natural polysaccharides have been widely used in developing protective coatings for bacteria. Meanwhile, they are often employed as bacterial carbon sources, such as inulin, β-glucan, and sodium alginate 47, 69, 70. However, carbon sources alone are insufficient to meet the holistic metabolic demands for the proliferation of bacteria. Ginger-derived exosomes enriched with polysaccharides, nucleic acids, proteins, and lipids have been assembled onto bacteria as versatile nutrient depots 71 (**Figure 6**). Inspired by the food-carrying strategy of ameba, Pan *et al.* prebound ginger-derived exosome-like nanoparticles (GELNs) to *Lacticaseibacillus rhamnosus* GG (LGG). Due to the close contact between LGG and GELNs, LGG can exclusively uptake GELNs despite the existence of other intestinal microorganisms. This nutrient-providing strategy has been demonstrated to elevate the LGG proliferation rate approximately 1.5 times under nutrient-deprived, competitive conditions. Nevertheless, supplementation of rich nutrients may support the growth of opportunistic pathogens. In this context, prebiotics, such as inulin, galacto-oligosaccharides, and xylo-oligosaccharides 2, which are preferentially utilized by beneficial microbes and confer measurable health benefits, can be employed for engineering bacteria. This rational approach can selectively enhance the competition capacity and fitness of engineered bacteria. Besides, nutrient-rich systems may inadvertently support opportunistic pathogens, and close contact between probiotic bacteria and the nutrient depot can help inhibit growth of opportunistic pathogens.

### 3.5 Microbial competition

In therapeutic settings, bacterial colonization is frequently constrained by ecological competition from microorganisms residing in the local microenvironment 72, 73. Exogenous strains must compete for nutrients, attachment sites, and host-derived resources. Meanwhile, disease-associated niches may be favorite for the expansion of pathobionts or opportunistic pathogens 74, 75. Strategies for relieving this dual competitive pressure for administered bacteria have been developed, including equipping exogenous bacteria with the capacity for selective support of their survival and persistence and restricting competing microbes, thereby improving their therapeutic performance 39, 76, 77. Xu *et al.* constructed an EPS-based hydrogel to create a local selective advantage for bacteria while suppressing competing pathogens. EPS-M76 in the hydrogel promoted the proliferation and metabolic activity of *Lactobacillus paracasei* TYM202, while lactic acid and acetic acid are released from the system to suppress the growth of pathogenic bacteria, thereby conferring a direct competitive advantage to the bacterial cargo 78. Oxygen regulation is another engineering route. Functionalized chlorella and *Bacillus subtilis* were encapsulated in a living microecological hydrogel. Sustained oxygen generation from chlorella reshaped the local oxygen distribution that favored bacterial proliferation while suppressing pathogenic bacteria. Therefore, differential oxygen responsiveness between beneficial and pathogenic microorganisms could be exploited for engineering other bacteria 79. Furthermore, competition can be shifted by creating an environment that selectively eliminates pathogens while preserving the bacterial payload. Wei *et al.* integrated a hydrogel containing peroxidase-like nanozymes with *Lactobacillus*. During hydrogel degradation in an infected vaginal microenvironment, *Lactobacillus* restored an acidic niche and provided H_2_O_2_, which was converted by the nanozymes into fungicidal ROS, thereby achieving selective killing of *Candida albicans.* Therefore, this approach preserved the bacterial viability and reshaped the vaginal microbiota toward a healthier state 80.

### 3.6 Antibiotic resistance

**Figure 7:**
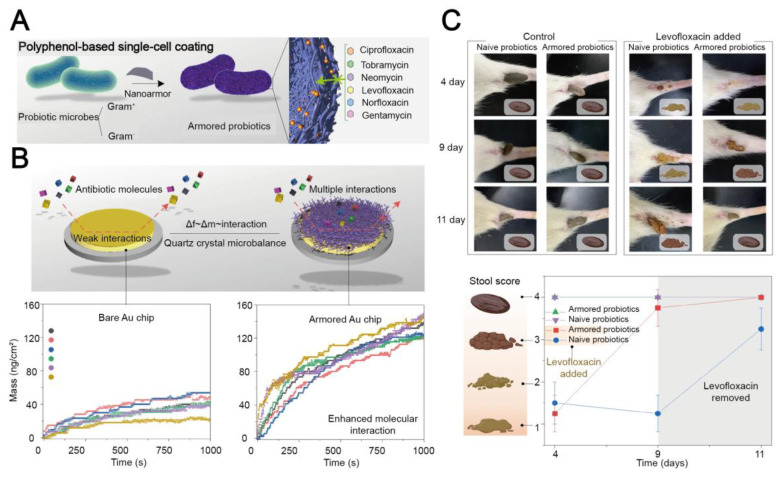
Representative examples of antibiotic-shielding engineering strategies. (A) Schematic illustration of a nano-armor that protects bacteria from a wide range of antibiotics with different molecular structures and properties. (B) Diagram for multiple interactions between polyphenol moieties and antibiotic molecules. (C) The severity of antibiotic-associated diarrhea (AAD) and dysfunction of the gastrointestinal tract assessed from the fecal samples and the stool scores. Adapted with permission from 82, copyright 2022, Springer Nature.

When bacteria are applied to infection-associated indications, such as severe wounds and gastrointestinal infections, antibiotic therapy is often used to rapidly suppress pathogen overgrowth. Although antibiotics remain indispensable in these settings, their non-specific bactericidal activity can disrupt commensal microbiota and inadvertently impair administered bacteria, thereby diminishing their colonization and compromising their therapeutic efficacy 81. Therefore, it is essential to protect bacteria from antibiotic-induced collateral damage when live biotherapeutics are deployed in conjunction with conventional anti-infective treatment. Current engineering strategies are designed to localize and inactivate antibiotics at the bacterial interface 82-85 (**Figure 7**). For example, a coating for bacteria was prepared from a biocompatible metal-phenolic network derived from tannic acid and Fe^3+^, which adsorbed and inactivated diverse antibiotic molecules through interfacial molecular interactions, thereby protecting engineered bacteria from collateral damage 83. This nano-armoring strategy reduced the detrimental impact of antibiotic therapy on exogenous bacteria and facilitated their subsequent colonization and proliferation.

### 3.7 Immune evasion

A significant impediment to the efficacy of bacterial therapy is rapid immune recognition and subsequent clearance of the administered bacteria, particularly, after systemic delivery such as intravenous injection. Rich conserved microbial molecular components, including lipopolysaccharides, peptidoglycan, flagellin, and CpG-rich DNA, are displayed on the bacterial surface. These components are detected by pattern-recognition receptors (PRRs), which promptly initiates inflammatory signaling, complement activation, and phagocytic elimination 86, 87. Therefore, immune surveillance can markedly compromise bacterial persistence and reduce their accumulation at target sites, thereby diminishing their therapeutic efficacy. For instance, intravenously administered *Salmonella Typhimurium* VNP20009 displayed unsatisfactory antitumor efficacy, even at the maximum-tolerated dose, which was supported by insufficient tumor accumulation at the tumor site 88. External surface engineering has become an important strategy to improve the performance of live bacterial therapeutics by masking pathogen-associated molecular patterns or camouflaging bacteria as host cells for immune evasion.

**Figure 8:**
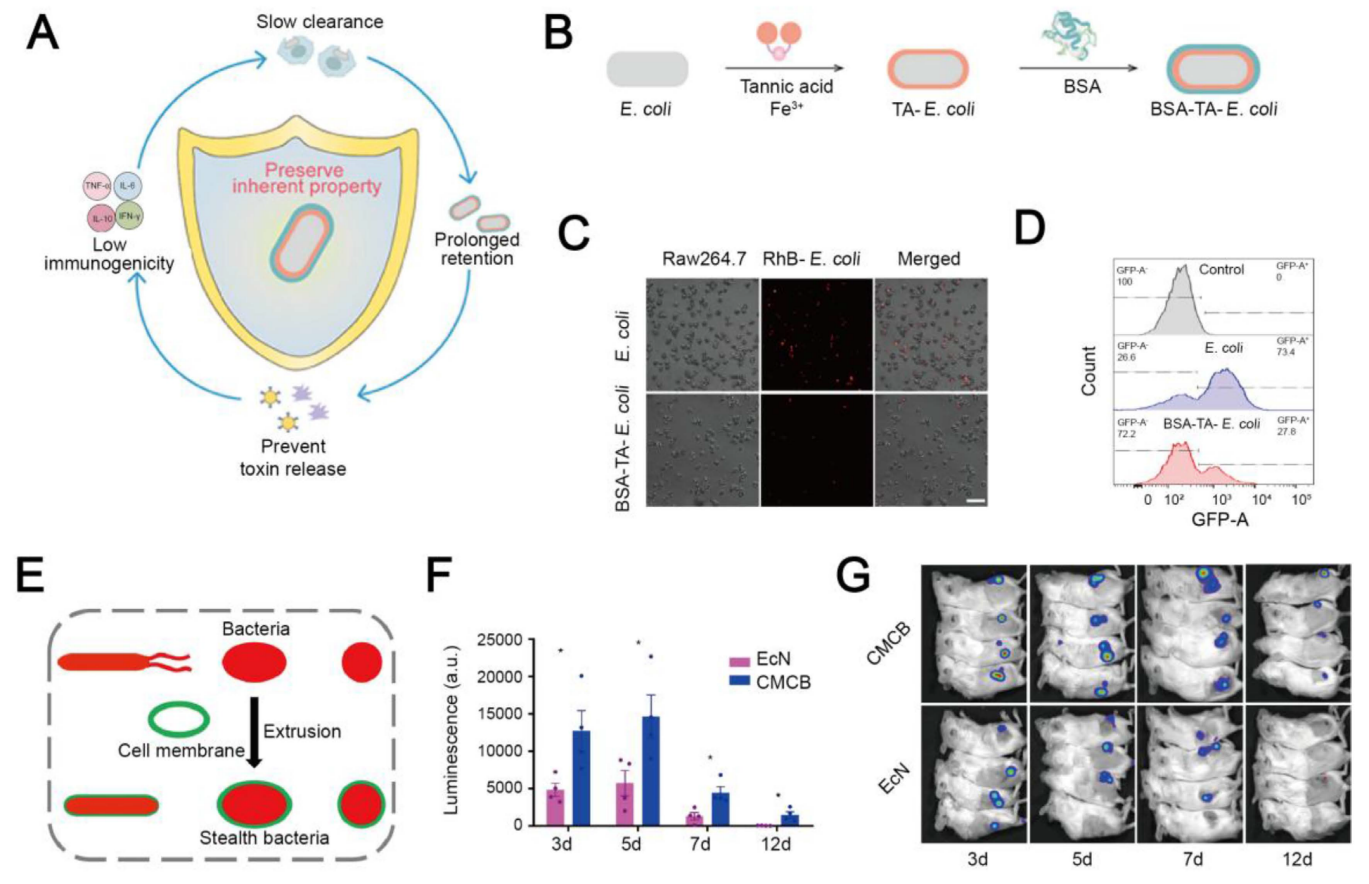
Representative examples of immune-evasive engineering strategies. (A-B) Schematic illustration of developing BSA-TA-*E. coli* that exhibits extended retention and enhanced biocompatibility *in vivo*. (C-D) Fluorescence images and flow cytometry plots confirm a significantly lower proportion of BSA-TA-*E. coli* is engulfed by macrophages compared to uncoated *E. coli.* Adapted with permission from 91, copyright 2025, Elsevier. (E) The process of preparing cell-membrane-coated bacteria (CMCB). (F-G) A bar chart and tumor images of the luminescence signal intensity in 4T1 tumor-bearing mice post-injection of EcN or CMCB. Adapted with permission from 93, copyright 2019, Springer Nature.

To minimize the recognition of bacterial surface antigens by immune cells in the body, an artificial shell can be directly coated on the bacterial surface. An inducible capsular polysaccharide system reported in a recent study helped the bacteria temporarily evade immune clearance and enhanced systemic delivery efficiency 89. In a FeTA-coated EcN system, an iron–tannic acid coating mitigated both complement-mediated and phagocyte-mediated bacterial killing, leading to a prolonged circulation time and a high tumor colonization level after intravenous injection 90. In another study, a tannic acid-based compact layer in conjunction with an albumin coating was used to mitigate endotoxin exposure, improving bacterial biocompatibility and reducing immune elimination 91. (**Figure 8A-D**) In addition, a polymeric stealth coating onto engineered *Lactobacillus acidophilus* protected the bacteria from macrophage recognition and phagocytosis, prolonging bacterial survival during the delivery process 92.

An alternative approach is to cloak bacteria with host cell membranes to present host-derived interfacial features during circulation. Camouflaging EcN by wrapping with red blood cell membrane lead to a 42-fold increase in tumor accumulation and a 14-fold prolongation in blood persistence compared with uncoated bacteria following intravenous injection. The encouraging results can be attributed to the membrane-derived anti-phagocytic properties, such as CD47-associated self-recognition signals 93 (**Figure 8E-G**). Collectively, these studies support that bacteria are rapidly eliminated by immune cells in the body because their native surfaces are exposed to complement proteins, phagocytes and innate immune receptors during systemic circulation. Bacterial surface engineering can improve their *in vivo* delivery efficiency and bacterial persistence by mitigating their early immune clearance.

### 3.8 Site-specific targeting and responsive release

The accumulation of sufficient bacteria at lesional sites is crucial for efficacious bacterial therapy. Therefore, site-specific targeting has become a critical attribute for bacteria. Targeting moieties can be introduced onto the surface of engineered bacteria to enhance their active targeting of specific cells or molecular signatures within inflamed or tumor tissues. In addition, external physical guidance strategies can be employed to direct engineered bacteria to diseased sites and improve their localized accumulation.

Diseased tissues often exhibit distinct physicochemical features, including gradients in pH, ROS, and glutathione (GSH), which can be exploited to achieve stimuli-responsive, site-specific release of engineered bacteria. Eudragit L100-55 is a commonly used pH-responsive polymer coating. The carboxyl groups in Eudragit L100-55 remain protonated under an acidic condition, thereby maintaining the integrity of the polymer during gastrointestinal transit. However, these groups become ionized at an intestinal pH, leading to polymer dissolution and localized release of bacteria 40, 94-96. To enable bacterial selective release at inflammatory lesions, a hyaluronic acid-based thiolated thioketal hydrogel was degraded to locally release *Lactobacillus reuteri* in response to excess ROS in the inflamed colon 97. In addition, a disulfide-rich self-assembling peptide coating on *Salmonella* underwent disassembly to facilitate local bacterial release in response to an elevated GSH level in a reductive TME 98.

Engineered bacteria can actively target disease sites via recognizing overexpressed cell surface-exposed and microenvironment-associated signal cues. The cues on the host cell surface are activated in response to pathological conditions, including aberrant cellular activation, epithelial injury, and membrane perturbation, leading to the upregulation of surface molecules that are accessible for microbial recognition. By decorating bacterial surfaces with appropriate targeting ligands, these interfacial changes can result in enhanced adhesion and retention at lesional sites. For instance, a fucoidan-based protective coating on EcN promoted bacterial localization to diseased mucosa through fucoidan-mediated binding to P-selectin, an endothelial adhesion molecule, as well as electrostatic adhesion to damaged epithelial surfaces. *In vivo* imaging results confirmed a 2.42-fold increase in the bacterial signal in the inflamed colon after administration of engineered EcN compared with bare EcN 57. Functionalization of the EcN surface with Annexin A5 was performed. The resulting engineered EcN recognized phosphatidylserine exposed on injured or apoptotic epithelial cells, thereby extending their lesional enrichment time up to 120 h 99. In addition, cell surface-directed localization was achieved by a hyaluronic acid-modified bacterial delivery system, and this system improved bacterial retention at CD44-enriched pathological sites 100, 101. Furthermore, active tumor targeting can be realized by conjugation of antibodies or aptamers onto the surface of engineered bacteria. These antibodies or aptamers can selectively recognize overexpressed proteins on tumor cells through ligand–receptor interactions 102-105. For instance, Geng *et al.* developed tumor-targeting engineered bacteria by conjugating AS1411, an aptamer, to *Salmonella Typhimurium* VNP20009 through amide condensation. The engineered bacteria exhibited a 4-fold increase in tumor accumulation at 60 h after intravenous administration, compared with their unmodified counterpartner 105.

Pathological lesions are often characterized by aberrant intercellular signaling and tissue remodeling, leading to localized accumulation of diffusible bioactive factors and extracellular enzymes. These soluble mediators can be exploited by engineered bacteria as cues for direct targeting or lesion responsive activation. For example, EcN with a surface IL-6 aptamer exhibited a 197.2-fold increase in the residence time than bare EcN in an inflamed colon tissue 106.

Spatiotemporal manipulation strategies have been developed to guide engineered bacteria to diseased sites by external physical stimuli, including a magnetic field, ultrasound, and light irradiation 107-111. For example, the *Magnetococcus marinus* strain MC-1 was guided toward solid tumors under an external magnetic field and then intrinsically migrated along an oxygen gradient toward hypoxic, necrotic tumor regions. Experimental data indicated that 55% of MC-1 cells successfully penetrated deep into hypoxic regions of colorectal cancers 107. A magnetically engineered bacterial strain was explored for localized anti-CD47 antibody release in another study 108. In this system, engineered bacteria carrying Fe_3_O_4_@lipid nanocomposites were guided to colon tumors via an external magnetic field. At the tumor site, the magnetic signals of Fe_3_O_4_ nanoparticles were converted into thermal signals. This magnetothermal effect subsequently induced bacterial lysis, leading to the release of therapeutic anti-CD47 antibodies, thereby enabling precise tumor immunotherapy.

### 3.9 Aerosol formulation

The pulmonary deposition level of inhaled bacteria is predominantly governed by the aerodynamic size of the bacteria-containing droplets or powder particles, rather than the size of individual bacterial cells. According to a coupled airway and mucus flow model, aerosols larger than 5 μm deposit primarily in the upper airways because of inertial impaction, whereas aerosols smaller than 1 μm may remain suspended in the air flow and they are then exhaled. By contrast, the deep lung delivery efficacy for aerosols is highest at the 1-5 μm size range 33. Accordingly, inhaled bacteria formulations for distal lung diseases should be prepared within a respirable aerodynamic size window. In alignment with this principle, Nicola *et al.* developed a spray dried Lactobacillus live biotherapeutic formulation at a mass median aerodynamic diameter of 2.4 μm, with approximately 74% below 3.3 μm, supporting its effectiveness in distal lung delivery 112.

For liquid aerosol delivery, Byun *et al.* compared the delivery efficiency via vibrating mesh nebulization and jet nebulization of LGG. They found that a higher percentage of the recovered dose was collected in the output filter when the vibrating mesh nebulizer was used, particularly for bacteria suspended in the MRS broth or PBS 34. For powder delivery, Nicola *et al.* showed that during spray drying with trehalose, leucine, sodium citrate and polysorbate, approximately 99% of the bacterial viability was retained 112. Tran *et al.* formulated spray freeze-dried LGG with lactose, mannitol and leucine and demonstrated that lactose protected the bacterial viability during dehydration, and the anti-*pseudomonal* activity was maintained in the optimized powder 35. However, viable LGG declined by approximately 1.8 log CFU/mL after three months of refrigerated storage despite preservation of LGG in a solid powder formulation.

It is noted that these engineering strategies are interconnected, because modification of the bacterial interface can lead to intertwined effects on stress resistance, adhesion, immune interactions, ecological competition, and metabolic fitness. From a synergistic perspective, surface coatings and encapsulating matrices can protect bacteria against multiple physiological and pathological insults, including gastric acidity and reactive oxygen species. Surface coverage may also reduce the exposure of immunogenic bacterial components and mitigate immune clearance 90. Multifunctionality can also be obtained from the intrinsic physicochemical properties of materials. For example, catechol rich polydopamine coatings can simultaneously enhance interfacial or mucosal adhesion and antioxidant protection, and they also provide an interface for the incorporation of additional functional components 113. By simultaneously alleviating several environmental stresses, these strategies can support bacterial viability, redox homeostasis, proliferation, and functional activity, thereby helping maintain metabolic fitness and therapeutic persistence *in vivo*. Nevertheless, these benefits may be accompanied by side effects. Thick or persistent coatings may mask native adhesins on the bacterial surface, mechanically impede bacterial growth and motility, and hinder the diffusion of nutrients, gases, metabolites, or secreted therapeutic molecules 114. Therefore, the composition, thickness, porosity, degradation kinetics, and responsive unmasking of the coating layer should be systematically optimized to overcome multiple biological barriers without impairing bacterial viability and functionality.

Multicomponent systems can provide distinct, complementary functions across different modules. For example, Zhou *et al.* coated *Limosilactobacillus reuteri* with Mn-doped CeO_2_ nanozymes and encapsulated the modified bacteria within alginate hydrogel microspheres 115. The nanozyme coating enhanced ROS scavenging, and the alginate microspheres improved bacterial survival under gastric acidic conditions and promoted delivery and retention of modified bacteria at inflamed colonic sites. This system promoted bacterial growth, meanwhile, it improved microbial homeostasis, epithelial integrity, nutrient absorption, and anti-inflammatory immunity, illustrating that rational multicomponent designs can address distinct biological barriers through complementary mechanisms. Although multiple modular integration can broaden the therapeutic functionality, the increasing system complexity may lead to different behavior of both the material platform and the bacterial cargo compared to a system with an individual component alone. The interactions between multiple components can influence bacterial fitness and therapeutic performance, and the complexity also results in challenges for manufacturing consistency, storage stability, and safety evaluation. Future studies should be conducted to reveal the interactions between these components in preparation, storage, administration, and release phases.

## 4. Design principles for hybrid systems of bacteria and materials

After the biological barriers for bacterial therapy have been discussed in the previous section, engineering principles to construct bacteria-material therapeutic systems are elaborated in this section. A range of engineering strategies have been developed, including surface modification, intracellular loading, and bulk encapsulation. Structural and interfacial features of bacteria are crucial for the implementation of these strategies because they govern their interaction with functional motifs and the surrounding biological environment (**Table 2**). In this section, we first delineate the fundamental structural characteristics of bacteria, with a particular emphasis on the bacterial surface, and then systematically review current engineering strategies for bacterial engineering. The successful construction of engineered bacterial therapeutics relies on efficient integration of functional materials with bacterial cells. This process is primarily governed by bacterial surface physicochemical characteristics, accessible functional groups, endogenous biosynthetic pathways, and the intracellular loading capacity 116-118. Therefore, a thorough understanding of bacterial structural features is essential for the rational design of engineering strategies and the development of functional bacterial therapeutics.

### 4.1 Structural characteristics of a bacterial surface

Bacteria possess a relatively simple, yet highly organized cellular architecture, comprising the cytoplasm, a nucleoid region, ribosomes, a cytoplasmic membrane, and an outer envelope structure. In Gram-positive bacteria, the outer envelope consists of a thick peptidoglycan cell wall enriched with teichoic acids and lipoteichoic acids. In contrast, Gram-negative bacteria have a thin peptidoglycan layer located between the periplasm and an outer membrane containing lipopolysaccharides. Many bacteria also carry additional surface-associated structures, such as capsules, flagella, pili, and other appendages, which influence the bacterial morphology, viability, adhesion, motility, colonization, and interaction with their surrounding environment.

**Figure 9:**
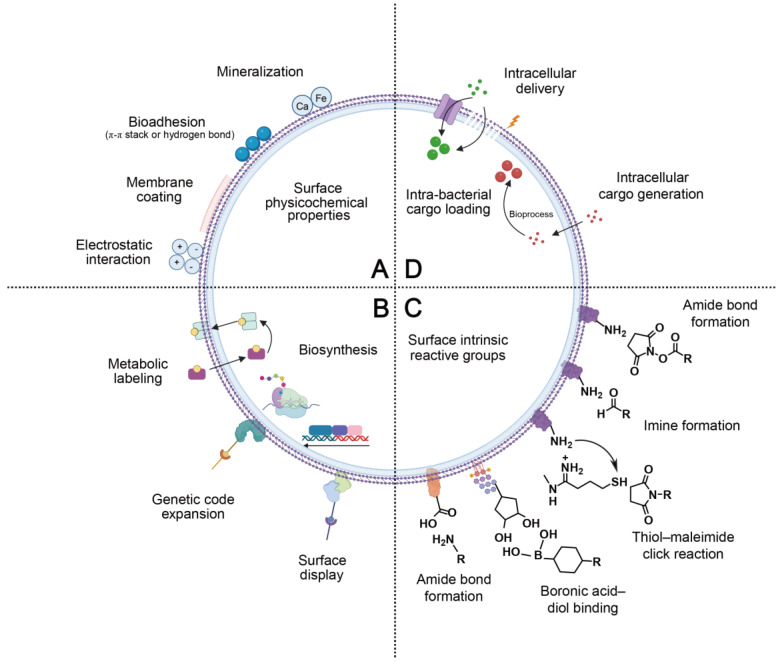
Schematic illustration of four principal approaches for bacterial engineering. (A) Surface modification through tuning physicochemical properties of the bacterial interface, including electrostatic interaction, membrane coating, bioadhesion, and biomineralization. (B) Biosynthesis-mediated modification through hijacking the endogenous bacterial biosynthetic machinery, such as metabolic labeling, genetic code expansion, and surface display. (C) Covalent conjugation to surface-intrinsic reactive groups, including amino, carboxyl, and cis-diol groups through amide bond formation, imine formation, thiol–maleimide click reaction, and boronic acid–diol binding, respectively. (D) Intra-bacterial cargo loading facilitated by cellular transporters, physical electroporation, and intracellular bioprocesses to strengthen their capacity to serve as carriers or *in situ* factories for therapeutic agents. Collectively, these strategies provide a versatile toolbox for engineering bacteria to enhance their stability, targeting capability, and therapeutic efficacy. Created in https://BioRender.com.

These externally exposed components provide the most accessible and chemically versatile interface for surface modification through biomaterial engineering, therefore, the bacterial surface has become the primary target for current bacterial engineering strategies (**Figure 9**). A variety of functional groups, such as carboxyl, amino, and cis-diol moieties, on surface-associated components 58, 119 provide tunable reaction sites for chemical modification of bacterial surfaces. Moreover, owing to the presence of abundant phosphate-containing components on bacterial surfaces, many bacteria exhibit an overall negative surface charge, which facilitates the adsorption of positively charged biomaterials through electrostatic interaction 45.

### 4.2 Negative surface charge-mediated electrostatic interaction

Owing to the presence of anionic interfacial components, including teichoic acids, lipoteichoic acids, lipopolysaccharides, and acidic polysaccharides, bacterial surfaces are generally negatively charged, enabling the adsorption of positively charged materials without prior chemical modification 120. This feature provides a straightforward and cytocompatible foundation for bacterial surface engineering via electrostatic interaction. For example, *Lactobacillus plantarum* with a negatively charged bacterial surface was coated with cationic polymer poly-L-lysine through electrostatic interaction 121. Building on the same principle, electrostatic deposition can be extended to layer-by-layer assembly through alternating deposition of oppositely charged materials. For instance, positively charged chitosan was first deposited onto *Bacillus coagulans*, followed by negatively charged alginate. The stepwise reversal of zeta potential after each deposition allowed the electrostatic formation of multilayers on the bacterial surface 122. A notable advantage of charge-mediated interaction lies in its mild aqueous-reaction condition, which is generally benign for maintaining the bacterial viability 123. The multilayer format offers a modular platform for additional surface functionalization. However, scale-up of this process remains challenging because of multiple repeated procedures of deposition, centrifugation and washing. Meanwhile, the formation of the resulting multilayer and their stability are sensitive to variations in pH and ionic strength, the deposition sequence, and the charge balance 124. In addition, when the surface charge approaches neutralization, these coated bacteria may be aggregated, thus impairing the coating homogeneity. Future efforts should be devoted to refining the process and ensuring the coating integrity under formulation-associated stresses, such as drying and reconstitution 123.

### 4.3 Membrane-like surface-mediated assembly coating

Bacterial envelopes present a soft, hydrated, negatively charged, and membrane-like amphiphilic interface. Therefore, exogenous lipids or membrane structures can adsorb, reorganize, and self-assemble on the bacterial surface under mild conditions, forming an additional membrane-like coating without repeated deposition or prior chemical activation 93, 120, 125. In this process, the assembled membrane serves as a physically stable outer cloak to improve bacterial viability and preserve their bioactivity. For example, EcN was coated with dioleoyl phosphatidic acid and cholesterol via simple vortex mixing for approximately 15 min, forming an additional self-assembled lipid membrane on the bacterial surface. This coating significantly improved bacterial survival against environmental stress and enhanced oral delivery efficiency and therapeutic efficacy in a murine colitis model 125. This principle can be further extended to biologically derived membrane structures. For instance, EcN was wrapped with erythrocyte membranes by mechanical extrusion to generate cell-membrane-coated bacteria with a distinct outer lipid shell, reducing their inflammatory responses, mitigating phagocytic clearance, and improving their *in vivo* persistence and target-site accumulation 93. The quality of membrane-based coatings is contingent upon the membrane source and the coating integrity. In addition, the coated bacteria remain sensitive to the lipid formulation and environmental conditions, such as the ionic milieu and the exposure to bile salts.

### 4.4 Chemically heterogeneous surface-mediated adhesive deposition

Adhesive deposition is a distinct strategy for bacterial surface engineering. Through interfacial adhesion, coating materials can be anchored onto bacterial surfaces. Bacterial envelopes exhibit a chemically heterogeneous interface characterized by multiple exposed functional groups, thus they serve as favorable substrates for adhesive deposition through hydrogen bonding, chelation, π–π stacking, and nonspecific interaction. In addition, covalent coupling can be applied to oxidized catechol-containing systems 120, 126, 127. Catecholamine-derived coatings, inspired by mussel adhesive chemistry, can undergo oxidative self-polymerization under a mild condition to form conformal surface layers with strong interfacial adhesiveness, thereby achieving adhesive deposition on bacterial surfaces 126, 127. For example, polydopamine nanoparticles have been deposited onto EcN to construct a living hybrid coating system for dopaminergic immunoregulation, thereby alleviating colitis 128. Interaction of polyphenols with metal ions to form a metal-phenolic system can be an alternative method for adhesive deposition onto bacterial surfaces. Phenolic components on the bacterial surfaces mediate interfacial anchoring. The resulting metal-phenolic coordination can stabilize the coating and endow the bacteria with additional protective or therapeutic functions 129. For example, the tannic acid/Fe(III) coordination was established on a bacterial surface to generate a single-cell nanoarmor. The transient protective shell enhanced bacterial resistance to antibiotics and improved therapeutic efficacy in antibiotic-associated diarrhea 82. The strong adhesiveness of these materials enables broad surface attachment and facilitates their application across diverse engineering contexts. However, the adhesive interaction may promote non-specific binding and interparticle crosslinking, which may lead to bacterial aggregation and heterogeneous surface coating. In addition, the performance of bioinspired adhesive deposition is highly dependent on pH, the precursor concentration, oxidation or coordination conditions, and assembly kinetics. These parameters should be systematically optimized to preserve bacterial viability and their native function, meanwhile, a broader and safer reaction window can be defined for controllable coating formation.

### 4.5 Nucleation-prone surface-mediated mineralization

Bacterial envelopes bear negatively charged and ion-interacting groups that can locally enrich mineral precursors, providing heterogeneous nucleation sites for inorganic particle growth. This feature has been used for surface mineralization and protective mineral shells can be generated on the surface of living bacteria under mild conditions. Beyond robust physical protection, mineral layers introduce novel physicochemical and biological activities of inorganic components to bacteria, thereby empowering new functionalities for bacteria, including pH responsiveness, catalytic reactivity, magnetic resonance responsiveness, bioactive ion release, and integration with other therapeutic modalities 48, 57, 130. For example, a pH-responsive biomineralized bacterial vaccine was developed by engineering recombinant *Bacillus subtilis* producing the HPV16 E7 antigen. In this vaccine, a calcium phosphate shell was formed on the bacterial surface to protect the bacteria 131. The calcium phosphate shell acted as a physical shield and displayed an acid-labile property in a gastric environment, thereby improving the survival of the bacteria during gastrointestinal transit, prolonging their intestinal retention, and enhancing their oral bioavailability. In addition, Ca^2+^ released from the mineral coating induced bile acid aggregation and mitigated gastrointestinal stress, ultimately improving the bacterial survival and augmenting therapeutic efficacy in a colitis model 130. Furthermore, an MnO_2_/Fe_2_O_3_ nanocoating was developed on a bacterial surface. The nanocoating served as a T1/T2 dual-mode MRI contrast layer for visualizing bacterial distribution in the gastrointestinal tract, and provided nanozyme-like antioxidant activity to scavenge ROS and alleviate inflammation 48.

From a translational perspective, surface mineralization offers the protection of bacteria against manufacture- and delivery-associated stresses, including oxygen exposure, ultraviolet irradiation, and ethanol treatment. Meanwhile, this strategy can be applicable to both obligate and facultative anaerobes. Despite these advantages, the effect of degradation products from the mineral layer on the local ionic environment and microbiota remains to be systematically evaluated. Meanwhile, a highly dense mineral shell may suppress bacterial proliferation and induce their transient dormancy, which should be robustly verified in *in vivo* models.

### 4.6 Surface intrinsic reactive groups for covalent modification

#### 4.6.1 Surface amino groups

Surface amino groups are among the most accessible native reactive sites on bacterial cells, and they are associated with surface proteins and molecular components on the cell wall. Owing to their nucleophilicity, these primary amines can be employed for covalent surface engineering under a mild aqueous condition, while preserving the bacterial viability. Amide bond formation is a fundamental amino-directed strategy. In this route, carboxyl-containing molecules are introduced either as preactivated N-hydroxysuccinimide (NHS) esters or activated carbodiimide, and these molecules are coupled to primary amines on the bacterial surface to form stable amide linkages. The major advantage of this strategy lies in the robustness of the resulting bond, which is favorable for persistent surface display during gastrointestinal transit and subsequent host interaction. For example, after the croconium dye was converted into an NHS ester, the ester group was directly conjugated to the amine group on the surface of engineered EcN, endowing the bacteria with photothermal activity while preserving the bacterial viability and maintaining the immunostimulatory function 132. A similar strategy was used to attenuate *Salmonella Typhimurium* VNP20009. The surface amine group was coupled with a carboxyl-containing JQ-1 derivative through 1-ethyl-3-(3-dimethylaminopropyl)carbodiimide (EDC)/Sulfo-NHS chemistry, generating a dual-drug-decorated bacterial biohybrid with enhanced tumor accumulation, photothermal ablation, and antitumor immune activation 133.

Primary amine groups on bacterial surfaces can also undergo amino-carbonyl coupling with aldehyde- or quinone-containing materials or molecules, forming imine-type covalent linkages 134-136. This strategy was applied to *Shewanella oneidensis* MR-1. Aldehyde-bearing ZIF-90/MB particles were covalently hybridized onto the bacterial surface via acid-labile imine linkages formed between the aldehyde groups and the amine groups. The resulting stable bacteria–material biohybrid preserved the bacterial activity and facilitated acidic environment-triggered dissociation 137.

Beyond direct covalent conjugation, native surface amino groups can be converted into secondary reactive moieties for the subsequent functionalization. A representative example is 2-iminothiolane-mediated surface thiolation. In contrast to direct cargo conjugation, this strategy enabled covalent anchoring of bacteria to biological substrates by creating a dynamic mucoadhesive interface. Primary amines on surface-exposed proteins in EcN were transformed into free thiols under cyto-compatible conditions. The introduced thiols underwent catalyst-free thiol-disulfide exchange with disulfide-rich mucin, resulting in tunable mucin attachment. This approach resulted in a 170-fold increase in mucosal adhesion of bacteria in mucin-enriched jejunum, ultimately improving therapeutic efficacy in jejunal mucositis 67. This design was subsequently extended to delivery of bacteria to the inflamed colon. Thiolated EcN showed enhanced mucus binding, prolonged intestinal retention, and improved restoration of microbiota homeostasis in inflammatory bowel disease 138.

#### 4.6.2 Surface carboxyl groups

Surface carboxyl groups are another type of native reactive sites present on bacterial cells. These groups are predominantly associated with peptidoglycan components and acidic residues in proteins exposed at the bacterial interface. Carboxyl groups are first activated by carbodiimides through EDC/NHS chemistry to generate amine-reactive intermediates for subsequent amide bond formation 139. This strategy has been used to attach amino-functionalized aptamers to EcN, including AS1411 for tumor targeting and an IL-6 aptamer for inflammation targeting. The modifications with AS1411 and the IL-6 aptamer improved bacterial localization at diseased sites and enhanced therapeutic efficacy in tumor-associated and colitis-associated disease models, respectively 105, 106.

#### 4.6.3 Surface cis-diol groups

Surface cis-diol moieties are also an important type of native reactive sites on bacterial cells. These groups are predominantly associated with polysaccharide-containing structures exposed at the bacterial interface. These motifs can reversibly interact with boronic acid-containing materials to form boronate ester linkages, thereby establishing a dynamic covalent bond, which is distinct from amino- or carboxyl-directed coupling bonds 140. The primary advantage of this strategy lies in its rapid assembly and environmental responsiveness, which enhances interfacial stabilization while retaining the capacity for stimulus-triggered dissociation or release. This strategy has been used to assemble artificial-enzyme-armed *Bifidobacterium longum* bacteria. The engineered bacteria exhibited enhanced intestinal targeting and retention, persistent ROS scavenging, and improved therapeutic efficacy in inflammatory bowel disease 58.

In general, the engineering strategies by harnessing the surface group of natural bacteria provide a flexible approach to constructing stable conjugation between bacteria and materials. However, these strategies have a few limitations. Because amino, carboxyl and cis-diol groups are widely distributed in bacterial surface proteins, cell wall components and polysaccharides, the modifications obtained are usually non-specific and may produce heterogeneous products. In addition, excessive coupling may undermine bacterial viability and mask natural adhesion molecules to impair bacterial movement or weaken the host-bacterial interaction.

### 4.7 Biosynthesis-enabled bacterial surface engineering

Bacteria undergo continuous remodeling of their surface structures through a biosynthesis process, such as glycans, proteins and polymers 141, 142. This process has been harnessed to reshape surface structures 143, 144. Both intrinsic components and novel functional molecules could be introduced into the bacterial outer surface for protection or functionalization 144, 145.

Metabolic labeling is a widely used strategy to functionalize bacterial surfaces. Exogenous, metabolically compatible precursor analogs can be processed by endogenous biosynthetic or remodeling machinery and their metabolic products are then incorporated into target surface structures during their formation or turnover 143, 146. In bacteria, this approach has been used for metabolically labelling diverse surface components, including peptidoglycan, capsular polysaccharides, surface glycans, and teichoic acids. This approach can be further extended to proteins when the translational machinery accepts noncanonical amino acid surrogates 147-151. For instance, D-alanine-derived precursors carrying oxaliplatin and a photosensitizer were simultaneously incorporated into peptidoglycan of tumor-targeting nonpathogenic *E. coli* MG1655, yielding a living platform for synergistic chemo-photodynamic therapy and immunogenic cell death induction 152. In addition, bioorthogonal reactive groups can be introduced for subsequent conjugation by this strategy. Ji *et al.* used this metabolic method to incorporate 3-azido-D-alanine into the cell wall of *Lactococcus lactis*. The introduction of the azide group facilitated a subsequent copper-free click reaction with dopamine- and β-glucan-based components, which enhanced gastrointestinal tolerance, intestinal retention, colonization, and antidiabetic efficacy 153.

**Figure 10:**
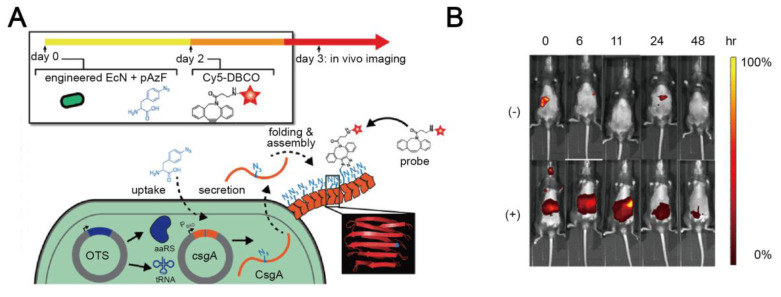
Representative examples of biosynthesis-enabled bacterial surface engineering. (A) The process of incorporating nonstandard amino acids into bacteria through an orthogonal translation system (OTS). These amino acids are targeted by an imaging probe. (B) Fluorescence imaging of live mice administered with either PBS (-) or engineered bacteria (+). Adapted with permission from 156, copyright 2018 American Chemical Society.

Orthogonal reactions can also be performed through genetic code expansion (GCE). In this approach, an orthogonal aminoacyl-tRNA synthetase/tRNA pair decodes a reassigned codon and a noncanonical amino acid is loaded into a growing peptide. Eventually, a reactive group is introduced at a predetermined residue of the target protein 145, 154, 155. Praveschotinunt *et al.* placed an in-frame UAG codon in csgA, a gene for the curli monomer CsgA of *E. coli*, and co-expressed an orthogonal aaRS/tRNA pair that reads UAG as p-azido-L-phenylalanine (pAzF) 156 (**Figure 10**). Once the curli fibers were assembled on the cell surface, the azide group on these fibers reacted with DBCO-Cy5 via copper-free click chemistry. Since the Cy5 label was stable *in vivo*, the engineered strain could be real-time tracked within the mouse gut by monitoring fluorescence signals.

Surface display strategies have been developed to expose specific proteins, peptide ligands or antigenic epitopes by reshaping bacterial surfaces. By genetically programming the expression, secretion, trafficking, and anchoring of recombinant proteins or peptide modules, these artificial proteins or peptides can be transported and presented on cell membranes, cell walls, spore coats or biofilm matrices 157-159. For example, Nguyen *et al.* genetically fused functional peptide domains into the C-terminus of CsgA, the major amyloid subunit of the *E. coli* curli system 160. After translation, the final fusion protein was secreted and then self-assembled into extracellular nanofiber networks. By fusing different peptide tags to CsgA, distinguished functionalities were achieved: a metal-binding domain for stainless steel adhesion, and an A3 peptide to template silver nanoparticles. Furthermore, Praveschotinunt *et al.* adapted this platform to EcN and produced curli fibers with a pro-repair cytokine trefoil factor 3 (TFF3) 161. Interestingly, the surface display strategy could also allow the incorporation of SpyTags for covalent capture of SpyCatcher-fused proteins, which resolves the issue of a large size for direct genetic fusion. For example, bacteria empowered through the surface display method by Zhang *et al.* remarkably alleviated colitis in a mouse model. *Lactococcus lactis* was engineered to display a SpyCatcher-AcmA fusion on the bacterial surface. In this system, AcmA acted as a cell-wall anchor. SpyTag-terminated polymers, such as polydopamine, were conjugated onto the surface through SpyTag/SpyCatcher covalent ligation. A dopamine shield was generated from this SpyTag/SpyCatcher complex to improve gastrointestinal stability, mucoadhesion, and local colonization, thus alleviating colitis in mice 162.

Biosynthesis-assisted surface engineering can introduce functional structural units in the process of surface remodeling, providing highly spatial control over the surface structure and stably displaying proteins, peptides or bio-orthogonal handles. However, their efficiency is convergent upon the metabolic activity, genetic operability and growth status of the bacterial chassis. In addition, genetic manipulation may raise concerns over the strain stability, biosafety and regulatory translation, which should be carefully addressed for their clinical application.

### 4.8 Intrabacterial cargo loading

In intrabacterial cargo loading, the intracellular space of bacterial cells serves as a protective compartment that shields functional cargoes from the surrounding milieu while preserving native surface structures involved in host interactions 163-165.

#### 4.8.1 Intracellular delivery

**Figure 11:**
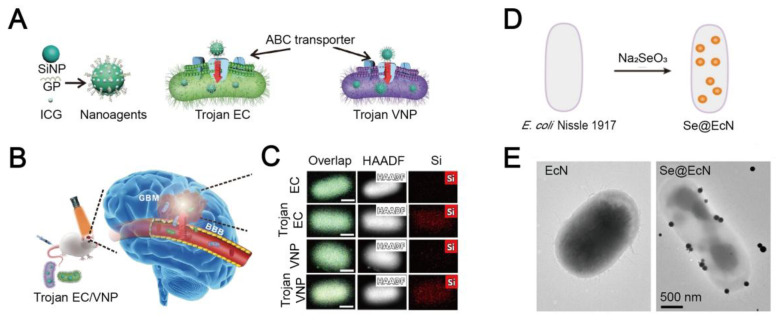
Representative examples of intrabacterial cargo loading. (A) Schematic illustration of the construction of a Trojan bacteria system. (B) Crossing a blood–brain barrier (BBB) and targeting and penetrating glioblastoma (GBM) tissues by the Trojan bacteria system. (C) High-angle annular dark field-scanning transmission electron microscope (HAADF-STEM) images confirm intracellular localization of silicon nanoparticles in a bacteria cell, rather than nonspecific adsorption on the bacterial surface. Adapted with permission from 165, copyright 2022, Springer Nature. (D-E) The preparation and characterization of Se@EcN. Adapted with permission from 169, copyright 2025, American Chemical Society.

The physicochemical properties of a cargo can facilitate its delivery into a cell via either native transport pathways or membrane permeabilization. For instance, silicon nanoparticles carrying indocyanine green (ICG) were modified with a glucose polymer, poly[4-O-(α-D-glucopyranosyl)-D-glucopyranose]. The resulting hybrid was internalized into attenuated *Salmonella typhimurium* or *E. coli* through an ATP-binding cassette (ABC) transporter. Since the envelope of both bacteria was undisturbed, the loaded bacteria successfully crossed the blood–brain barrier (BBB) and delivered ICG into the glioblastoma tissue 165 (**Figure 11A-C**). For cargos that cannot enter the bacterial cytoplasm through native transporters, permeabilization strategies, such as electroporation, offer an alternative loading route for a diverse range of cargoes. Zoaby *et al.* discovered that electroporation can improve the efficiency in loading doxorubicin-containing liposomes into bacteria 166, 167. It is noted that the permeabilization process must be meticulously tuned to preserve the viability and the motility of the bacteria since the pores created through the process for the cargo entry may be detrimental to the bacterial cells.

#### 4.8.2 Intracellular cargo generation

Inspired by the biosynthetic and redox machinery of bacteria, an *in situ* cargo generation strategy has been developed. After an appropriate soluble precursor is introduced into the culture medium and enter a bacterial cell, the bacterial intracellular compartment can serve as a reactive microenvironment to generate the cargo with therapeutic function as well as a storage compartment for the generated cargo 168. For example, bacterial cells internalize soluble selenium precursors and reduce them to selenium nanoparticles, which in turn scavenge ROS and amplify the therapeutic effect 49, 169 (**Figure 11D-E**). Combined metabolomic and proteomic analyses revealed that nanoSe biosynthesis was associated with metabolic remodeling, with enhanced redox adaptation and TCA cycle activity, but reduced carbohydrate catabolism and oxidative phosphorylation 49. It is noteworthy that this process relies on a continuous precursor supply, and the supply may not be feasible *in vivo*. The biosynthesized product may be exported across the envelope, leading to an unforeseen biological consequence 170.

Intracellular cargo loading is a very useful strategy when bacterial cells are expected to act as live carriers to carry agents while preserving their outer structures and biological functions. The transporter-mediated loading method is relatively mild, and it can maintain the integrity of bacterial cell membranes. Therefore, it is suitable for applications that requires bacterial movement, tissue penetration or crossing barriers, such as tumor-targeting treatment. In contrast, permeability-based loading can accommodate a broad loading content, but this method should be meticulously optimized to prevent compromising the viability and hindering the motility of bacteria. *In situ* cargo generation of functional materials in cells is achieved by harnessing the metabolic process of bacteria, thus providing self-protection or therapeutic advantages under pressure conditions. However, this strategy is heavily contingent upon the availability of precedents, the metabolic activity of bacteria and the retention of products, which may impede its applicability in the human body. Meanwhile, metal precursors or uncontrolled particle formation may promote ROS generation and elevate the metabolic burden, which may compromise the genome integrity and increase the cost of selecting biomineralization traits. In contrast, controlled synthesis may detoxify soluble metal species and produce ROS scavenging nanomaterials, such as SeNPs. These context-dependent outcomes warrant further evaluation of bacterial growth performance and their long-term functional stability 170.

### 4.9 Bacterial engineering methods based on bulk encapsulation

Beyond engineering bacteria by harnessing their interfacial components and introducing new chemical functionalities, bulk encapsulation strategies have been developed to incorporate multiple bacteria cells and therapeutic agents within a biocompatible matrix. Bulk encapsulation methods not only provide collective protection against adverse environmental conditions prior to reaching target sites but also facilitate bio-responsive release of bacteria and therapeutic agents 49, 64, 171-175. Encouragingly, the bulk encapsulation process is significantly more simplified compared to the modification of single bacteria cells. This process can be readily scaled up for large-scale production, exhibiting greater potential for clinical translation 176. An array of techniques has been reported to encapsulate multiple bacterial cells as a population within one matrix to support their growth and proliferation. For instance, Luo *et al.* encapsulated EcN in a chitosan–alginate gel matrix via CaCl_2_ cross-linking 177; IIomuanya *et al.* developed mucoadhesive PVA/PCL electrospun scaffolds for intravaginal delivery of *Lactobacillus fermentum*, metronidazole, and sodium cocoamphoacetate to manage bacterial vaginosis 178; and Liu *et al.* encapsulated lactic acid bacteria in a 3D-printed biocompatible living hydrogel 179. Furthermore, Yang *et al.* co-encapsulated EcN and indole-3-propionic acid in dextran (Dex)-TA hydrogel microspheres via droplet microfluidics, confirming this multifunctional delivery system was more effective in disease treatment than bacteria-only microspheres 180.

Compared with single-cell surface engineering, encapsulating bacteria in hydrogels, fibers, microcapsules, or other protective matrices is much simpler and more scalable. However, the control of the behavior of individual bacterium during the bulk encapsulation process is often very challenging. In addition, matrix design should strike a balance between protection of bacteria and their access to the surrounding microenvironment, because the encapsulation of bacteria within these matrices may impede the diffusion of nutrients and toxic metabolites, hinder the migration of bacteria, and ultimately diminish the treatment efficiency. Therefore, bulk encapsulation can be considered for scalable and protective formulation, but careful balance should be tailored between matrix protection, encapsulation efficiency, and bacterial viability.

## 5. Engineered bacteria in therapeutic applications

**Figure 12:**
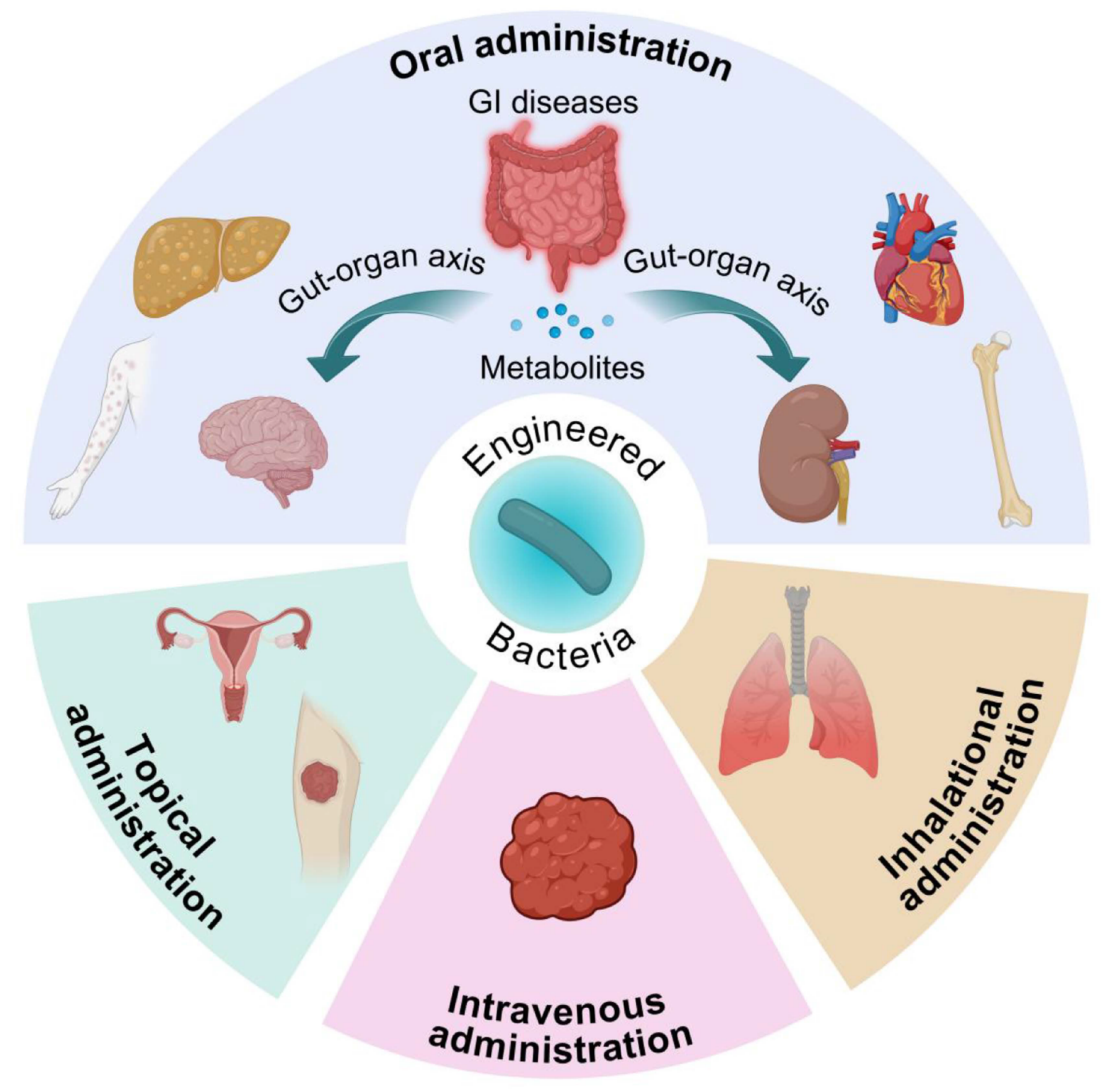
An overview of four administrative routes for engineered bacteria in therapeutic application. Oral administration of engineered bacteria not only allows the treatment of gastrointestinal (GI) diseases but also the extension of systemic therapeutic effects through multiple gut–organ axes via the production of beneficial metabolites. Topical administration is predominantly used for the treatment of vaginitis and wound healing. After intravenous administration, engineered bacteria home to tumor sites and exert their antitumor activity. Inhalational administration has shown promise for the treatment of pneumonia and lung cancer. Created in https://BioRender.com.

Despite multiple physiological and pathological barriers that hinder the survival and impair biological function of administered bacteria, recent engineering strategies have substantially improved their viability and functional performance during transport to diseased tissues. In this section, we summarize recent progress in the therapeutic application of engineered bacteria via different administration routes (**Figure 12**).

### 5.1 Oral engineered bacteria

The bioengineering-assisted approach for oral delivery of bacteria overcomes the physicochemical, biological, and ecological barriers encountered during gastrointestinal transit, improving their survival and colonization. By enhancing the stability and prolonging the functional persistence of bacteria in the gut, this approach not only augments bacterial efficacy in gastrointestinal disorders but also extends their therapeutic potential to diseases in distant organs through the gut-organ axis 181-186. For example, Yang *et al.* coated EcN with tannic acid and mucin to obtain EcN@TA-Ca^2+^@Mucin 65. These engineered bacteria displayed enhanced ROS-scavenging activity owing to the abundant phenolic hydroxyl groups in tannic acid, and the mucin coating improved bacterial colonization through multiple interactions with the intestinal mucus layer. Together, these acquired properties from tannic acid and mucin extended the colonic retention time of the bacteria to 96 h and improved their therapeutic efficacy in a murine colitis model. To target the gut microbiota–trimethylamine–trimethylamine N-oxide axis in atherosclerosis, Chen *et al.* developed nanoparticle-functionalized bacteria by loading fluoromethylcholine into nanoparticles and conjugating these nanoparticles onto polydopamine-coated LGG, yielding PDMF@LGG 182. The polydopamine coating improved intestinal retention, while the methyl thioethanol ester in nanoparticles scavenged ROS and triggered responsive release of fluoromethylcholine in the gut. These engineered bacteria were administered to elevate the abundance of the *Lachnospiraceae* and *Oscillospiraceae* family to ameliorate dysbiosis, ultimately lowering the circulating trimethylamine N-oxide level and attenuating atherosclerotic progression in mice. Besides, oral bacteria can function as a vaccine delivery vehicle. Xia *et al.* developed a biomineralized *Bacillus subtilis* system to sustainably produce HPV16 E7 antigen* in situ*. Notably, the calcium phosphate mineral coating facilitated a 4-fold increase in intestinal retention of the bacteria compared with uncoated ones, while sustaining the antigen persistence over 4 days. This engineered bacterial vaccine systematically activated CD8^+^ T cells, resulting in the suppression of 64% of TC-1 tumors 131.

### 5.2 Topical engineered bacteria

Topical administration of bacteria is increasingly explored in the treatment of cutaneous and mucosal inflammatory disorders, particularly, wound healing and vaginitis. Under these chronically infected microenvironments, physicochemical alterations, pathogen colonization and sustained inflammation compromise the bacterial fitness and diminish their therapeutic efficacy. To overcome these barriers, bacteria can be encapsulated within protective biomaterials to enhance their viability and facilitate their targeted colonization at disease sites. For instance, Liu *et al.* engineered *Lactobacillus rhamnosus* CLK101 (LRh) to biosynthesize antioxidant selenium nanoparticles and then encapsulated the engineered bacteria within a phospholipid polymer hydrogel to prepare gel@F-LRh 49. The hydrogel network mimicked a three-dimensional ECM structure, promoting bacterial metabolic activity and stability. The resulting gel@F-LRh effectively inhibited the proliferation of pathogenic *Staphylococcus aureus* and scavenged excess ROS in diabetic wounds, accelerating the healing of infected wounds in a mouse model. Zhang *et al.* developed a *Bacillus subtilis*-containing hydrogel platform, B. sub@HMHA, for the treatment of fungal vaginitis 187. This living hydrogel locally produced high-molecular-weight hyaluronic acid and antimicrobial peptides, thereby reducing inflammation and inhibiting fungal growth. For bacterial vaginosis, bacterial therapy must overcome two major barriers: poor mucosal retention and intense competition with pathogenic microbiota. To address these issues, Ilomuanya *et al.* developed mucoadhesive electrospun scaffolds for intravaginal delivery of *Lactobacillus fermentum* together with metronidazole 178. The engineered bacteria showed improved vaginal retention with a mucoadhesion value of 57.86% in a mouse model. Meanwhile, the co-delivered metronidazole was expected to suppress pathogenic bacteria, thereby creating a more favorable niche for *Lactobacilli* colonization and restoring the vaginal microbial balance. However, this combined strategy faces an important challenge: locally administered antibiotics may impair the efficacy of therapeutic bacteria. Since topical antibiotics are commonly used for biofilm-associated diseases such as infected wounds and vaginitis, future efforts should be devoted to protecting live therapeutic bacteria while maintaining antimicrobial efficacy against pathogens.

### 5.3 Intravenous engineered bacteria

Upon systemic administration, various live bacteria, including *Salmonella typhimurium*, EcN and *Bifidobacterium*, can intrinsically migrate to solid tumors due to their hypoxia tropism. However, the majority of systemically administered bacteria are rapidly cleared by the mononuclear phagocyte system, resulting in a low tumor colonization level and limited therapeutic efficacy 88. Furthermore, compromised anti-tumor immune responses are often seen in an immunosuppressive TME, characterized by nutrient deprivation and accumulation of toxic metabolites 13. Therefore, to improve bacteria-based tumor treatment outcomes, their tumor accumulation can be enhanced through evasion of immune clearance, and immune activation in the TME can be realized through their combination with complementary therapies. To improve tumor accumulation of intravenously delivered bacteria, efforts have been made to reduce systemic clearance and enhance tumor targeting. To reduce immune clearance, Cao *et al.* coated EcN with erythrocyte membranes to generate cell membrane-coated bacteria (CMCBs) 93. The membrane coating facilitated a reduction in the immunogenicity of bacteria and the phagocytic uptake, while maintaining the bacterial viability. Although intravenously administered CMCBs majorly accumulated in liver and spleen at 1 h post-injection, the number of bacteria within healthy organs gradually decreased over time. Notably, this approach resulted in an approximately 42-fold increase in tumor accumulation compared to uncoated EcN post murine tail vein injection. To enhance tumor targeting, Xiao *et al.* covalently coupled a monomethyl auristatin E (MMAE)-based aptamer-drug conjugate to *Salmonella typhimurium* VNP20009, yielding VNP@Sgc8c-MMAE 104. The intrinsic tumor tropism of the bacteria in combination with aptamer-mediated targeting resulted in less biodistribution in the liver, spleen and kidney, but a 3-fold increase in tumor accumulation and remarkable promotion of cytotoxic T cell infiltration in a Miapaca-2 tumor xenograft model.

### 5.4 Inhalational engineered bacteria

Living bacteria via pulmonary administration can modulate local microbial population and induce inflammatory disturbance in lung diseases, but its effectiveness is convergent upon preserving bacterial activity during the preparation and delivery process and engineering the formulation to overcome pulmonary barriers. In bacterial pneumonia, Fu *et al.* coated living Lactobacillus rhamnosus with chitosan, hyaluronic acid and ononin to generate OASCLR particles at approximately 1.32 μm in length and 0.51 μm in width 38. The coating enhanced bacterial stability, facilitated ROS scavenging and promoted hyaluronic acid-mediated interaction with CD44-expressing inflammatory macrophages. Within OASCLR, living Lactobacillus rhamnosus retained its ability to produce lactic acid and inhibit Staphylococcus aureus. In murine models of Staphylococcus aureus pneumonia, nebulized OASCLR reshaped the lung microbiota and attenuated pulmonary inflammation. Nicola *et al.* developed a pulmonary formulation for chronic obstructive pulmonary disease using three live Lactobacillus strains, L. plantarum BAA 793, L. acidophilus 4356 and L. rhamnosus 53103 in combination with trehalose, leucine, sodium citrate and polysorbate. They prepared the powder formulation via spray drying 112. In the resulting powder, approximately 99% of the bacterial viability was retained. The aerodynamic profile of the powder was applicable for distal lung delivery, with a mass median aerodynamic diameter of 2.4 μm and approximately 74% below 3.3 μm. In mouse models of chronic obstructive pulmonary disease, such as emphysema induced by porcine pancreatic elastase, with or without additional lipopolysaccharide-associated inflammation, administration of the spray dried formulation through an Insufflator resulted in a reduction in the alveolar enlargement and airway resistance, a decrease in the levels of MMP-9, neutrophil elastase, C reactive protein and IL-8, and an increase in bronchoalveolar lavage IgA. Interestingly, Nicola *et al.* also showed that prolonged pulmonary persistence of inhaled live Lactobacillus may not be essential for the therapeutic activity. Although whole lung colony counts of the administered three-strain *Lactobacillus* blend steadily decreased over 72 h, the local L(+) lactic acid level remained relatively stable. These findings suggest that transient bacterial residence may be sufficient to reshape the local microbial population and generate sustained exposure to bioactive metabolites. Therefore, an effective metabolic exposure window and a clearance profile of inhaled living bacterial therapeutics should be carefully defined.

## 6. Conclusion and perspectives

After the intricate relationship between microbial homeostasis and human health has been gradually unveiled over the past decades, bacteria have emerged as a promising candidate for living therapeutics for a wide spectrum of diseases. Bacterial therapeutics have been investigated across multiple administration routes, including oral, topical, intravenous, and inhalational delivery. However, these bacterial administration routes have encountered *in vivo* barriers, such as exposure to low pH and ROS, nutrient limitation, poor adhesion, competition from microorganisms within the body, immune clearance, and unsatisfied targetability. By addressing these challenges individually or in combination, engineering strategies can improve the survival, retention, and biodistribution of therapeutic bacteria, enhance immunomodulatory effects, and promote microbial homeostasis across various disease contexts, ultimately improving therapeutic efficacy. Nevertheless, although several *in vivo* barriers are shared among different administration routes, the feasibility and relevance of corresponding engineering strategies vary substantially depending on tissue-specific microenvironments and disease contexts. For instance, nutrient-supplying strategies have been validated in gastrointestinal bacterial delivery, but their potential value in other administration routes remains underexplored, despite nutrient availability representing a fundamental requirement. Moreover, strategies that improve bacterial adhesion after oral administration may not be applicable to other delivery routes, suggesting that the transferability of an effective engineering design across disease models requires further evaluation. Of note, these findings have largely emerged from preclinical studies across diverse disease models, and their clinical relevance remains to be further validated.

To achieve optimized bacterial therapy, future development should begin with rational selection and engineering of bacterial chassis. Strain-specific differences in the metabolic preference, the surface structure, colonization, stress resistance, and the immunomodulatory capacity, can have a substantial impact on biomaterial interactions and therapeutic outcomes 188. A deep comprehension of the innate characteristics of individual bacterial cells is essential for biological engineering of these beneficial bacteria. In addition, efforts should be directed towards the design of genetically modified bacteria as living treatment factories, which offer additional advantages compared to non-modified ones in the field of disease treatment. Recent advances in synthetic biology, including programmable gene circuits and clustered regularly interspaced short palindromic repeats (CRISPR)-based editing, may further enable engineered probiotics to sense disease-associated signals, regulate therapeutic output, and improve biosafety through controllable functional modules. Beyond therapeutic potency, strain selection should also account for growth stability, surface characteristics, stress tolerance, and production reproducibility, as these properties directly influence subsequent material modification and clinical translation.

Beyond strain selection, biomaterial design should consider bacterial chassis, material interfaces, and pathological environments as an interconnected design space, and the design space can be optimized to improve therapeutic outcomes. Within this framework, integration of different materials onto bacteria may provide complementary properties and synergistic effects, while the incorporation of these materials may introduce the analytical and manufacturing complexity, and they may have an impact on biosafety and translational feasibility. Looking ahead, artificial intelligence (AI) guided material design may provide a new paradigm for developing biomaterials compatible with living bacteria. Moving beyond the empirical adaptation of existing materials, AI could help navigate the multidimensional relationships among interfacial properties, bacterial fitness, host compatibility, therapeutic efficacy, and manufacturability. This capability may facilitate the identification of material architectures that reconcile competing requirements and guide the rational integration of complementary components into synergistic systems tailored to specific disease contexts.

Although biomaterial engineering can substantially enhance bacterial delivery performance and therapeutic efficacy across multiple dimensions, clinical translation of bacterial therapeutics is governed by developability, manufacturability, and regulatory compliance in addition to functional performance in preclinical models. Bacterial therapeutic products with clinical potential must be produced from a reproducible, scalable manufacturing process, maintain stability throughout storage and administration, and meet predefined quality attributes consistent with Good Manufacturing Practice (GMP) and regulatory evaluation. From a translational perspective, bulk encapsulation does not require strain-specific modification of individual bacterial cells, therefore, it is applicable to a broad range of bacterial strains 189. Its intrinsic limitation, however, arises from mass-transfer constraints. As the matrix thickness increases, the nutrient diffusion efficiency declines while toxic metabolites accumulate in the matrix core, resulting in reduced cell viability in the central region of large constructs and a small viable population concentrating at the periphery 114. Therefore, the size and geometry of the matrix should be designed to balance between bacterial loading and the survival of cells in the interior of the matrix. Electrostatic adsorption and layer-by-layer assembly offer considerable design flexibility, but the multilayer assembly is sensitive to the ionic strength, deposition sequence and charge balance. This process also requires repeated deposition–centrifugation–washing cycles, complicating the reproducible scale-up process. The aggregation of coated bacteria may compromise the coating homogeneity as the surface charge approaches the neutrality. Membrane-based coatings can be applied to the bacterial surface through a comparatively simple process, such as coextrusion or sonication. The operational complexity is significantly reduced, while their translation raises distinct quality-control concerns. For synthetic lipid coatings, the batch-to-batch consistency may be compromised due to the lipid composition, the oxidation state and the membrane fluidity, whereas for cell-derived membranes, the donor source, the purification procedure, compositional variations, and residual biological impurities become the principal risks for manufacturing reproducibility and storage stability 190. Covalent modification yields stable, site-defined functionalization of the bacterial surface, yet the coupling reagents and over-derivatization can impair the cell viability and function in a dose- and time-dependent manner. The covalent reaction conditions should be mild and they should be validated via functional-activity assays 176. Adhesive deposition and controlled mineralization build a protective shell in few steps under mild conditions, but uncontrolled polymerization or coordination may lead to uneven coating and aggregation. Therefore, release testing should be conducted to determine the coating content, the percentage of aggregates, residual ions, and the mineral loading content, together with the dissolution kinetics. Biosynthesis-assisted surface engineering relies on the bacterial own biosynthetic machinery, the degree of functionalization depends on metabolic and expression activity and is highly sensitive to culture conditions and growth state, leading to batch-to-batch variability in effective ligand density. Intrabacterial cargo loading also faces challenges in increasing the loading efficiency and preventing payload leakage during storage and prior to use.

In addition to manufacturing concerns, long-term preservation is critical for maintaining bacterial viability, functionality, and practical applicability. For example, the freeze-drying preservation method is one of promising methods for stabilizing bacteria. However, drying, freeze-thaw, and prolonged storage may damage bacterial cell membranes and inactivate bacterial proteins, thereby diminishing bacterial viability. Although cryoprotectants and lyoprotectants help preserve bacterial viability 191, preservation-associated stresses may compromise the integrity of the engineered material interface as these stresses may disrupt electrostatic coatings, detach adsorbed lipid membranes, alter covalent conjugates, dissolve mineralized layers, or impede the release from encapsulating matrices. In the freeze-drying product, the bacteria should remain viable, meanwhile the coating structure, load stability, and the therapeutic function should be retained.

Equally important, the stability of the bacterial progeny should also be systematically examined 192. The effects of prolonged culture and storage on bacterial cell division, genetic stability, phenotypic consistency, and functional fidelity should be systematically evaluated in working cell banks, as these attributes are critical for the safety and efficacy of bacterial therapeutics 193. A stable therapeutic function after prolonged bacterial proliferation is essential to maintain the batch-to-batch reproducibility and long-term efficacy. Therefore, functional consistency after serial passages should be considered as an important quality control indicator.

Beyond manufacturing and preservation, another critical aspect of clinical translation is the characterization of pharmacokinetics and pharmacodynamics of engineered bacterial therapeutics, as these properties have a direct impact on the exposure response relationships underlying their efficacy and safety. Unlike a static molecular entity, a bacterial therapeutic is a dynamic population that may proliferate, die, mutate, exchange genetic materials, or alter its function in response to the host environment. Therefore, while an administered dose may be informative, it is insufficient to determine the resulting exposure. A meaningful assessment should distinguish the administered nominal dose, the viable dose at the target site, and the functionally active fraction during the treatment period. Strain identity testing, combined with culture-based enumeration and strain-specific qPCR or ddPCR can be applied to route-appropriate samples, such as feces, blood, local swabs, respiratory specimens, or tissue biopsies, to confirm the administered strain and quantify the viable and total bacterial abundance at the sampled sites. Meanwhile, serial sampling can be employed to characterize the persistence of colonization at the target site and the kinetics of its eventual clearance. A key limitation, however, is that the sampled material may not adequately represent the bacterial state in the region of interest. Feces, for example, reflect the distal lumen rather than the mucosal community. Quantification is complicated by the viable-but-non-culturable (VBNC) state, in which bacteria remain alive yet fail to grow under standard conditions 194, whereas DNA-based qPCR/ddPCR methods cannot distinguish live from dead bacteria 195. Moreover, measurement of bacterial metabolites offers a functionally informative readout, but this measurement cannot be used to determine the abundance of bacteria. For example, butyrate, a short-chain fatty acid produced by commensal bacteria, induces colonic regulatory T-cell differentiation and ameliorates colitis 196. Fecal butyrate levels have been proposed as a biomarker of treatment efficacy in fecal microbiota transplantation 197. However, host absorption of the metabolite may deviate the measured level from the actual production level by bacteria. In addition, time-dependent drift from ongoing microbial metabolism after sampling and non-standardized measurement methods pose a compounded challenge to interpreting the metabolite data.

Pharmacokinetics and functional monitoring are also essential for evaluating effective elimination or functional silencing of engineered bacteria after completing their therapeutic action. This is particularly important because prolonged *in vivo* persistence after therapeutic treatment may lead to excessive colonization, ectopic migration, unintended dissemination, or opportunistic pathogenicity 70, 198. Therefore, beyond post-treatment monitoring, the fate of engineered bacteria should be considered from early stages of product development. Programmable clearance mechanisms and functional silencing circuits should be incorporated during system design to enable controlled elimination or deactivation when treatment is completed 199.

**Figure 13:**
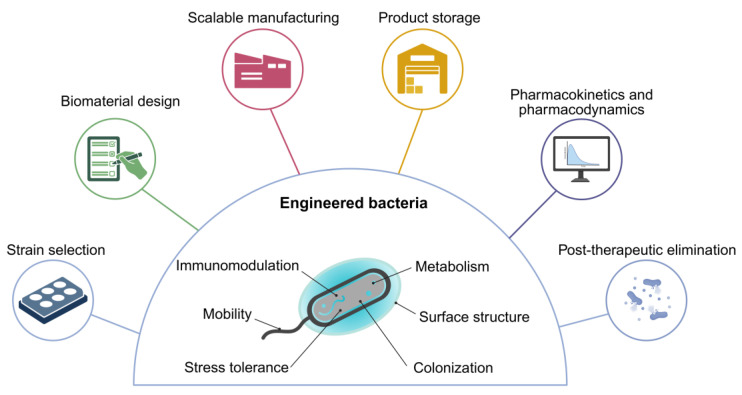
Challenges for clinical translation of engineered bacteria. Created in https://BioRender.com.

To address the aforementioned challenges, comprehensive and sustained collaborative efforts are essential among researchers in the fields of microbiology, synthetic biology, biomaterials science, process development, manufacturing-associated science and technology, pharmaceutics, and clinical research. Through interdisciplinary efforts, engineered bacteria could enter clinical translation to offer broad therapeutic benefits for patients across a wide range of disease conditions (**Figure 13**).

## Figures and Tables

**Table 1:** Probiotic treatment in clinical trials

Routes	Probiotics	Indications	Phase	ID numbers
Oral	*B. breve*	Crohn’s disease	Not applicable	NCT04842149
	*B. longum*		Not applicable	NCT00305409
	*L. acidophilus; L. rhamnosus*		Not applicable	NCT00374374
	*L. acidophilus; L. rhamnosus*	Ulcerative colitis	Not applicable	NCT00374725
	*L. plantarum*		Phase 2/3	NCT01193894
	*EcN*		Phase 4	NCT01772615
	*P. freudenreichii*		Not applicable	NCT02488954
	Engineered *L. lactis* to secret IL-10		Phase 2	NCT00729872
	Lactobacillus; Streptococcus; Bifidobacterium	Necrotizing enterocolitis	Not applicable	NCT06118801
	*L. reuteri*	Gingivitis	Not applicable	NCT06234839
	*L. reuteri*	Periodontitis	Not applicable	NCT04478643
	*L. rhamnosus; B. longum*	Dental caries	Not applicable	NCT03078179
	*S. salivarius*	Halitosis	Not applicable	NCT07384130
	*B. lactis*	Respiratory tract infections	Not applicable	NCT04122495
	*L. rhamnosus*	Anxiety	Not applicable	NCT03427515
	*B. bifidum*	Attention-deficit/Hyperactivity disorder	Phase 3	NCT04958460
	*Lactobacillus*	Epilepsy	Phase 2	NCT05539287
	*Bifidobacterium; Lactobacillus; Enterococcus*	Parkinson's disease	Phase 4	NCT04871464
	*L. paracasei; L. plantarum; L. rhamnosus; L. helveticus; B. breve*	Alzheimer's disease	Phase 1	NCT06181513
	*Lactobacillus*	Breast cancer	Not applicable	NCT03290651
	*Bifidobacterium; Lactobacillus; Lactococcus*	Atherosclerosis	Not applicable	NCT03100162
	*L. salivarius; B. breve*	Atopic dermatitis	Phase 3	NCT01500941
	*L. acidophilus; B. lactis*	Acne	Phase 4	NCT05216289
	*L. rhamnosus*	Vaginal dysbiosis	Not applicable	NCT05796921
	*L. rhamnosus; L. reuteri*	Bacterial vaginosis	Phase 4	NCT03894813
	*Lactobacillus*	Polycystic ovary syndrome	Phase 2	NCT04029805
	Engineered EcN to convert oxalate to formate	Hyperoxaluria	Phase 1	NCT04629170
	Engineered EcN to reduce methionine levels	Homocystinuria	Phase 1	NCT05462132
	Engineered EcN to express PAL and LAAD	Phenylketonuria	Phase 1/2	NCT03516487
	Engineered *L. lactis* to secrete proinsulin and IL-10	Type 1 diabetes	Phase 1/2	NCT03751007
	Engineered EcN to convert ammonia to L-arginine	Hyperammonemia	Phase 1/2	NCT03447730
Topical	Engineered *L. lactis* to secrete human TFF1	Oral mucositis	Phase 2	NCT03234465
	*B. subtilis; B. clausii*	Vaginitis	Not applicable	NCT06165354
	*Lactobacilli*	Bacterial vaginosis	Not applicable	NCT00752193
	*L. reuteri*	Wound healing	Not applicable	NCT03210779
	Engineered *L. lactis* to secret FGF-2, IL-4 and CSF-1		Phase 2	NCT06111183
	Engineered *L. reuteri* to secret CXCL12		Phase 2	NCT05608187
Inhalational	L. lactis	Chronic rhinosinusitis		NCT04048174
	L. sakei			NCT05427695
	L. casei; L. rhamnosus			NCT03587545
	L. lactis	COVID-19		NCT04458519
IT injection	Engineered EcN to express c-di-AMP	Solid Tumors and Lymphoma	Phase 1	NCT04167137
IV injection	Engineered *B. longum* to express IL-12	Solid Tumors	Phase 1	NCT04025307

L. acidophilus: Lactobacillus acidophilus; L. rhamnosus: Lactobacillus rhamnosus; L. plantarum: Lactobacillus plantarum; L. reuteri: Lactobacillus reuteri; L. salivarius: Lactobacillus salivarius; Lactobacilli, Lactobacillus species; L. lactis: Lactococcus lactis; L. sakei: Lactobacillus sakei; L. casei: Lactobacillus casei; B. breve: Bifidobacterium breve; B. longum: Bifidobacterium longum; B. lactis: Bifidobacterium lactis; B. subtilis: Bacillus subtilis; B. clausii: Bacillus clausii; EcN: Escherichia coli Nissle 1917; P. freudenreichii: Propionibacterium freudenreichii; S. salivarius: Streptococcus salivarius; PAL: phenylalanine ammonia lyase; LAAD: L-amino acid deaminase; TFF1: trefoil factor 1; FGF-2: fibroblast growth factor 2; IL-4: interleukin-4; CSF-1: colony-stimulating factor 1; CXCL12: C-X-C motif chemokine ligand 12; c-di-AMP: cyclic di-adenosine monophosphate; IL-10: interleukin-10; IL-12: interleukin-12.

**Table 2:** Representative bacterial modification strategies: advantages and limitations

Strategy	Bacteria/materials	Result	Advantages	Limitations	Refs.
Electrostatic interaction	*L. plantarum*/poly-L-lysine	6.86 ± 0.12 log CFU/mL of viable cells in SGF	Mild reaction conditions	Bacterial aggregation; repeated deposition and washing	121
	*B. coagulans*/chitosan-alginate multilayer	4-fold increased GI survival *in vivo*			122
Membrane coating	EcN/cholesterol lipid coating	2-fold higher gastric survival *in vitro*	Simple process	Membrane source; quality control	125
	EcN/erythrocyte membrane coating	14-fold longer blood retention post injection			93
Adhesive deposition	EcN,* E. coli BL21* /TA-Fe^III^ nanoarmor	12.56 × 10^6^ CFU/g feces with levofloxacin	Universal adhesion	Bacterial aggregation	82
	EcN/PDA nanoparticles	5 to 10 times higher viability in SGF			128
Mineralization	EcN/MnO_2_-Fe_2_O_3_-CaCO_3_ shell	17-fold increase of survival in SGF	Robust protection	Impaired bacterial viability; metal safety concern	48
	*B. fragilis*/CaCO_3_ shell	5-fold survival in intestine *in vivo*			130
	Recombinant *Bacillus subtilis*/carboxymethyl chitosan coating	Cecum retention time exceeds 72 h			131
Covalent modification	VNP20009/AS1411 aptamer	4-fold higher tumor enrichment post injection	Stable conjugation	Impaired bacterial viability and functions	105
	*B. longum*/single-atom catalyst	2-fold higher intestinal retention			58
Intracellular loading	VNP20009/glucose polymer + ICG silicon nanoparticles	Targeting glioblastoma tissue	Surface structure preservation	Premature leakage	165
	EcN/intracellular Selenium nanoparticles	Macrophage modulation in colitis			169
Metabolic labeling	*E. coli* MG1655/D-Ala-OXA and D-Ala-PPa	Peak tumor accumulation at 12 h post injection	Mild reaction conditions; precise modification	Genetic instability; culture-condition sensitivity	152
	Engineered *L. lactis*-GLP-1/dopamine-β-glucan shield	20,666-fold higher GI survival *in vitro*			153
Genetic code expansion	EcN/pAzF bioorthogonal handle	*In vivo* intestinal tracking			156
Surface display	EcN/CsgA-TFF curli display	Local TFF delivery in gut			161
	Engineered *L. lactis* displaying SpyCatcher/SpyTag polydopamine	3-fold higher retention in the GI tract			162
Bulk encapsulation	EcN/chitosan-alginate matrix	12 h survival time in SGF	Simple process; broad strain applicability	Restricted nutrient and waste exchange	177
	*Lactobacilli.*+metronidazole /PVA/PCL nanofibres	57.86 ± 0.95% vaginal mucoadhesion *in vitro*			178
	*L. bulgaricus*/3D-printed hydrogel	Controlled bacteria release			179
	EcN+IPA/dextran-TA microspheres	4-fold higher intestinal retention			180

CFU: colony forming unit; SGF: simulated gastric fluid; EcN: Escherichia coli Nissle 1917; TA-Fe^III^: tannic acid–iron(III); PDA: polydopamine; MnO_2_: manganese dioxide; Fe_2_O_3_: ferric oxide; CaCO_3_: calcium carbonate; IL-15: interleukin-15; ROS: reactive oxygen species; OXA: oxaliplatin; PPa: pyropheophorbide-a; GI: gastrointestinal; GLP-1: glucagon-like peptide-1; pAzF: p-azido-L-phenylalanine; NSAA: nonstandard amino acid; CsgA: curli-specific gene A; TFF: trefoil factor; ICG: indocyanine green; IPA: indole-3-propionic acid; PVA: polyvinyl alcohol; PCL: polycaprolactone.

## Abbreviations

FAO/WHO: Food and Agriculture Organization of the United Nations/World Health Organization
LGG: Lacticaseibacillus rhamnosus GG
IBD: inflammatory bowel disease
AMPs: antimicrobial peptides
SCFAs: short-chain fatty acids
ROS: reactive oxygen species
TLR2: Toll-like receptor 2
TME: tumor microenvironment
NK cells: natural killer cells
DCs: dendritic cells
PD-L1: programmed death-ligand 1
EcN: Escherichia coli Nissle 1917
E. coli: Escherichia coli
NHS: N-hydroxysuccinimide
EDC: 1-ethyl-3-(3-dimethylaminopropyl)carbodiimide
GCE: genetic code expansion
aaRS/tRNA: aminoacyl-transfer RNA synthetase/transfer RNA
pAzF: p-azido-L-phenylalanine
DBCO: dibenzocyclooctyne
Cy5: cyanine 5
DBCO-Cy5: dibenzocyclooctyne-cyanine 5
TFF3: trefoil factor 3
BBB: blood–brain barrier
ICG: indocyanine green
ATP: adenosine triphosphate
ABC transporter: ATP-binding cassette transporter
PELA: poly(ethylene glycol)-polylactide
Dex: dextran
TA: tannic acid
mGN: carboxymethylated β-glucan
Fh: ferrihydrite
GSH: glutathione
PRRs: pattern-recognition receptors
IL-6: interleukin-6
CD44: cluster of differentiation 44
CD47: cluster of differentiation 47
HPV16: human papillomavirus type 16
LRh: Lactobacillus rhamnosus CLK101
ECM: extracellular matrix
HMHA: high-molecular-weight hyaluronic acid
CMCBs: cell membrane-coated bacteria
MMAE: monomethyl auristatin E
PTT: photothermal therapy
CDT: chemodynamic therapy
PDT: photodynamic therapy
SDT: sonodynamic therapy
HMME: hematoporphyrin monomethyl ether
BiL: Bifidobacterium longum
ICD: immunogenic cell death
STING: stimulator of interferon genes
MRI: magnetic resonance imaging
